# Supplementary material for: A Cognitive Neural Architecture Able to Learn and Communicate through Natural Language
Source: PLoS One. 2015 Nov 11;10(11):e0140866. doi: 10.1371/journal.pone.0140866 (PMC4641699; doi:10.1371/journal.pone.0140866)
Supplement: S1 Appendix — (PDF) [file pone.0140866.s001.pdf]

Appendix 1 Examples from the learning sessions

The following conventions are used when typing phrases and/or commands through the interface:

- only lowercase letters are used, with no punctuations, no special characters; uppercase letters are used only for the first character of proper nouns; sentences do not start with a capital letter (unless they start with a proper noun);
- by convention, questions starts with a question mark, as: “*? how old are you”;*
- *w*ords with suffix are split in the form: base -suffix; e.g. animals → animal -s, writing → write -ing, etc.

1 First example: verbs and personal pronouns

In this example the system should combine the use of some verbs with that of personal pronouns.

The teacher can start by typing the two phrases:

the personal pronoun for a male person is he

the personal pronoun for a female person is she

then he should type phrases as

Susan is a female name

Susan is a doctor

Susan is drive -ing the car

Tim is a male name

Tim is a student

Tim is read -ing a book

Elisabeth is a female name

Elisabeth is a secretary

Elisabeth is write -ing a letter

Max is a male name

Max is an actor

Max is go -ing to the theater

…

and other similar phrases with different names. The order in which the phrases are submitted is not important, and they can be mixed with other kinds of phrases.

Now the teacher can ask the question

? what is Max do -ing

starting with a question mark, without other punctuations, and following the rule discussed previously for words with suffixes. Then he can suggest target word groups and target phrases that lead to the correct answer. Since he would like that the system uses the personal pronouns, the first part of the output should be the word "he", which can be obtained through the following word-group extractions and associations:

.word_group Max

.phrase Max is a male name

.word_group male

.phrase the personal pronoun for a male person is he

.word_group he

The word group obtained at the end of this exploration phase is the first part of the output. It should be rewarded, however the system should be warned that the output phrase is not complete. The command that the teacher should use in this case is

.partial_reward

The only difference between the partial reward and the complete reward is that the first is terminated by a CONTINUE action, while the latter is terminated by a DONE action.

To complete the answer, the teacher can suggest the following word-group extractions and associations:

.word_group Max

.phrase Max is go -ing to the theater

.word_group is go -ing to the theater

The last word group is the second and final part of the desired output, therefore it should be rewarded:

.reward

Now the teacher can test if the system is able to answer to similar questions, as for instance:

what is Tim do -ing

.exploitation

The system will answer:

he is read -ing a book

Note that it is not necessary to train the system with an example using a female name: the system will be able to use correctly both personal pronouns according to the gender. For instance, if the teacher asks the question:

? what is Elisabeth do -ing

the system will answer correctly, being able to use correctly both personal pronouns according to the gender.

2 Second example: categorization

This example uses the animal classification to show the categorization ability of the system. A first very simple test can be made by launching the program and typing phrases as:

the turtle is a reptile

the eagle is a bird

the dog is a mammal

...

(all lowercase, without punctuations) mixed to other phrases, as for instance

fish -es live in the water

reptile -s have cold blood

the turtle is slow

…

The order in which the phrases are submitted is not important. The teacher could now ask the system to tell him an animal belonging to one of the categories that he used before, e.g.

tell me a mammal

Clearly at this point the system has no idea of the meaning of this phrase, because it started from a blank condition (*tabula rasa*). However, it can use this phrase to start an exploration phase, during which the system can retrieve phrases memorized by the association mechanism and build new phrases through partially-random action sequences. The teacher can suggest to the system a target phrase or a

target word group. The exploration process is terminated when it produces the target phrase / target word group, or when the number of iterations becomes greater than a predefined limit.

For instance, if the teacher types the command:

.word_group mammal

the system starts an exploration phase, which terminates when the target word group "mammal" is extracted from the working phrase buffer. Therefore, the command:

.phrase the dog is a mammal

starts another exploration phase that is terminated when the working phrase, which is retrieved from the word group through the association mechanism, becomes equal to the target phrase. At this point, the teacher can type the command:

.word_group dog

The system will start a new exploration phase, which terminates when the target word group "dog" is extracted from the working phrase buffer. The word group corresponds to a good output, so the teacher can reward the system using the command

.reward

During the reward phase, the system retrieves the state-action sequence that led to a good output. The association between each state of the sequence and each corresponding action is rewarded by changing the connection weights of the state-action association SSM through the DHL rule.

Finally, the teacher can ask the system to say an animal belonging to a category different from the one used for training it, e.g.

tell me a reptile

and start the exploitation operating mode through the command

.exploitation

At the end of the exploitation phase, the system will respond with a correct output. If the question is repeated, the system will answer with another correct answer, generally different from the previous one.

This test, as well as several other tests used in the cross validation, show that the ANNABELL system is able to learn that the "is a" couple is used in phrases as "the dog is a mammal" to state that a concept belongs to a category, and that the "tell me a" group in a question can be used for asking to

retrieve a concept from a category. There are two key features of the ANNABELL system that are particularly important for this type of abstraction.

- The connections that are affected by the reward (i.e. the connections of the state-action association SSM) are connected to action neurons, rather than being directly connected to output words or phrases. In this way, the system learns preferentially to build the output through sequences of elementary operations on word groups or phrases.
- The input of the state-action association SSM includes equal-words vectors. In the previous example, when the system retrieves the phrases associated to the word group “a reptile”, it will recognize that the phrase “the lizard is a reptile” is more appropriate than other similar phrases (as for instance “the raven is a bird”) because the last word (“reptile”) is equal to the second word of the word group and to the last word of the input phrase (“tell me a reptile”), as in the association used in the training example (“a mammal” with “the dog is a mammal”).
