# Supplementary material for: A Cognitive Neural Architecture Able to Learn and Communicate through Natural Language
Source: PLoS One. 2015 Nov 11;10(11):e0140866. doi: 10.1371/journal.pone.0140866 (PMC4641699; doi:10.1371/journal.pone.0140866)
Supplement: S2 Appendix — (PDF) [file pone.0140866.s002.pdf]

Appendix 2 Example from CHILDES database

From CHILDES database, Warren-Leubecker corpus, file david.cha.

1 Comparison between ANNABELL system (left) and real child (right)

ANNABELL system

Participants:
- SYS, Ann, ANNABELL system;
- TEA, Teacher.

*TEA: Ann, why do not you tell, tell Dad about you playing Pac_Man .
*SYS: Michael brought me to the bumpers junction .
*SYS: I played Pac_Man .
*TEA: did you win ?
*SYS: yes, I won .
*TEA: did you play other games ?
*SYS: I played Space_Invaders .
*TEA: did you win ?
*SYS: no, I did not win .
*TEA: how many games did you play ?
*SYS: I played :
*SYS: Space_Invaders, one ;
*SYS: Pac_Man, two ;
*SYS: Donkey_Kong, three ;
*SYS: three games .
*TEA: I know what is better to talk about .
*TEA: you need to tell them about jumble junkman .
*SYS: jumble junkman will be my letter man .
*TEA: how about Harry hair ?
*SYS: Harry hair will be Bob's letter man .
*TEA: tell me what Mum put on your shirt .
*SYS: a sticker .
*TEA: a sticker .
*SYS: a dress_for_doll .
*TEA: ok .
*SYS: a wheel .
*TEA: right .
*SYS: a necklace .
*TEA: a necklace .
*SYS: a shoestring .
*TEA: a shoestring .
*SYS: a ring .
*TEA: a ring .
*SYS: a bracelet .
*TEA: right .
*SYS: a j@l .
*TEA: what kind of j@l was that ?
*SYS: a capital j@l .

Real child

Participants:
- CHI, David, target child, 5 years 10 months;
- MOT, Mother.

*MOT: Christopher , why don't you tell , tell Daddy about you playing Pac_Man .
*MOT: didn't Michael take you to play Pac_Man at bumper's junction ?
*MOT: huh ?
*CHI: yeh .
*MOT: well ?
*MOT: did you win ?
*MOT: what did you do ?
*CHI: I won !
*MOT: how many games did you play ?
*CHI: &um , I don't know .
*MOT: you don't know ?
*MOT: I know what's better to talk about .
*MOT: you need to tell (th)em about , &um , junk+man .
*CHI: xxx .
*MOT: your letter man !
*CHI: no !
*MOT: yes !
*MOT: tell (th)em what I put all over your shirt .
*MOT: &um &um .
*CHI: sticker .
*MOT: sticker .
*CHI: and a dress for a Barbie doll , and a wheel , and part of a necklace , and a shoestring , and a ring , or whatever .
*MOT: a bracelet .
*CHI: a bracelet .
*CHI: a key , a coat+hanger .
*MOT: and what's on the back ?
*CHI: &um .
*MOT: a piece of paper .
*CHI: piece of paper .
*CHI: and i@l , and a j@l , and a pencil , broken , and a button , and a chain , and a ring , and a crayon , and a peanut , two j@l-s +...
*MOT: how about those safety+pins ?
*CHI: I don't see (th)em .
*MOT: all those safety pins that are holding the things on your shirt .
*CHI: safety pin .
*CHI: string , string , string , string .

*TEA: how about those safety_pins ?
*SYS: Mum used some safety_pins to hold the things on the shirt .
*TEA: so, when will you wear your letter man dress ?
*SYS: May the twelfth .
*TEA: I think you wear it May the third ?
*SYS: no May the twelfth .
*TEA: May the twelfth ?
*SYS: yes .
*TEA: tell me the names of the letter men .
*SYS: a@l achoo .
*TEA: achoo ?
*TEA: and what, how about b@l ?
*SYS: b@l beautiful buttons .
*TEA: and tell me the rest of them .
*SYS: c@l cotton candy .
*TEA: okay .
*SYS: d@l delicious donuts .
*TEA: oh, I like that .
*TEA: okay .
*SYS: e@l exercise .
*TEA: right .
*SYS: f@l funny feet .
*TEA: oh, like mine .
*SYS: g@l gooey gum .
*TEA: very good .
*SYS: h@l Harry hair .
*TEA: right .
*SYS: i@l itchy itch .
*TEA: okay .
*SYS: j@l jumble junkman .

*MOT: and what kind of j@l is that ?
*CHI: capital .
*MOT: and what kind is that ?
*CHI: a lower case .
*MOT: a lower case , that's right .
*MOT: so when are you gonna wear that to school ?
*MOT: May the twelveth [: twelfth] , I think .
*CHI: I think we wear it May the third .
*MOT: no , it's May the twelveth [: twelfth] .
*MOT: is everybody in class gonna be wearing something ?
*CHI: mhm .
*MOT: well what are the other lettermen , letter people that there's gonna be ?
*MOT: tell me some of the names of the letter people .
*CHI: a@l .
*MOT: and what's a@l stand for ?
*CHI: achoo .
*MOT: achoo ?
*MOT: and what , how (a)bout b@l ?
*CHI: beautiful buttons .
*MOT: and , tell me the rest of them .
*CHI: c@l is cotton candy .
*CHI: d@l is delicious donuts .
*MOT: oh I like that .
*MOT: and how about c@l ?
*CHI: I already told you about c@l !
*MOT: you did ?
*CHI: &uh huh , cotton candy .
*MOT: oh , that's right .
*CHI: and e@l , exercise .
*MOT: okay .
*CHI: and +...
*MOT: f@l .
*CHI: f@l , funny feet .
*MOT: oh !
*MOT: like mine .
*CHI: but they're big !
*MOT: oh , okay .
*CHI: and +...
*MOT: g@l .
*CHI: gooey gum .
*MOT: gooey gum , alright .
*CHI: and , h@l , Harry hair .
*MOT: I .
*CHI: scratchy scratch .
*MOT: no , that's not for i@l .
*MOT: is that itchy something ?
*CHI: itchy itch .
*CHI: and +...
*MOT: and you got j@l , and what'd you say that was ?
*CHI: jumble junk+man .
*MOT: jumble junk+man .

2 Declarative sentences used to build the experience of the system in a text-based virtual environment

###
you are at the bumpers junction
Michael brought you here
you play Space_Invaders
you do not win
you play Pac_Man
you win
you play Donkey_Kong
you do not win
you leave

###
you are at school
you can see the teacher your classmate -s
the teacher says
next week we will put on a show about the letter -s of the alphabet
each of you will play a letter man
your mum will help you to prepare the dress for your letter man
you will wear your letter man dress May the twelfth
the letter men are
a@l achoo
b@l beautiful buttons
c@l cotton candy
d@l delicious donuts
e@l exercise
f@l funny feet
g@l gooey gum
h@l Harry hair
i@l itchy itch
j@l jumble junkman
Susan your letter man will be the achoo
Antony your letter man will be beautiful buttons
Andy your letter man will be cotton candy
Nicole your letter man will be delicious donuts
Carol your letter man will be exercise
Helen your letter man will be funny feet
Matt your letter man will be gooey gum
Bob your letter man will be Harry hair
Chris your letter man will be itchy itch
Ann your letter man will be jumble junkman

##
you are at home
you can see Mum
#Mum says
we have to prepare the dress for your letter man jumble junkman
we can put many thing -s all over your shirt
#
Mum put -s a sticker on your shirt
Mum put -s a dress_for_doll on your shirt
Mum put -s a wheel on your shirt
Mum put -s a necklace on your shirt
Mum put -s a shoestring on your shirt
Mum put -s a ring on your shirt
Mum put -s a bracelet on your shirt
Mum put -s a j@l on your shirt
Mum has done put -ing things
the j@l is a capital j@l
Mum use -s some safety_pin -s to hold the thing -s on the shirt

##
you are at the central park
Dad brought you here
you play hide_and_seek
you do not win
Susan win -s
you play bowl -s
you win
you play frisbee
you do not win
Oliver win -s
you play tag
you win
you leave

##
you are at school
you can see the teacher your classmate -s
the teacher says
Ann your desk mate will be Susan Dale
Matt your desk mate will be Bob Fingers
Helen your desk mate will be Carol Davis

##
you are in Susan 's room

you can see Susan aunt Carol
aunt carol says
Susan you must tidy up your room
#
Susan put -s a doll in her toy_box
Susan put -s a doll_house in her toy_box
Susan put -s a doll_dress in her toy_box
Susan put -s a toy_horse in her toy_box
Susan put -s a toy_dog in her toy_box
Susan put -s a puppet in her toy_box
Susan has done put -ing things
the puppet is a white cat
Susan place -s some book -s in the bookshelf

###
Ann you will celebrate your fifth birthday February the seventh

###
the fantastic four are
Mister Fantastic
#Reed Richards
The Invisible Woman
#Susan "Sue" Storm
The Thing
#Ben Grimm
The Human Torch
#Johnny Storm

###
the seven kings are
first Romulus
second Numa Pompilius
third Tullus Hostilius
fourth Ancus Marcius
fifth Tarquinius Priscus
sixth Tarquinius Superbus
seventh Servius Tullius

3 Training sentences

*TEA: tell me about you play -ing bowl -s
*SYS: Dad brought me to the central park
*SYS: I play -ed bowl -s
*TEA: ? did you win

*SYS: yes I won
*TEA: ? did you play other game -s
*SYS: I play -ed hide_and_seek
*TEA: ? did you win
*SYS: no I did not win
*TEA: ? how many game -s did you play
*SYS: I play -ed
*SYS: hide_and_seek one
*SYS: bowl -s two
*SYS: frisbee three
*SYS: tag four
*SYS: four game -s

*TEA: tell me about Susan Dale
*SYS: Susan Dale will be my desk mate

*TEA: tell me about Bob Fingers
*SYS: Bob Fingers will be Matt's desk mate

*TEA: tell me what Susan put in her toy_box
*SYS: a doll
*SYS: a doll_house
*SYS: a doll_dress
*SYS: a toy_horse
*SYS: a toy_dog
*SYS: a puppet
*TEA: ? what kind of puppet was that
*SYS: a white cat
*TEA: ? how about those book -s
*SYS: Susan place -ed some book -s in the bookshelf

*TEA: Ann
*TEA: ? when will you celebrate your fifth birthday
*SYS: February the seventh
*TEA: ? February the sixth
*SYS: no February the seventh
*TEA: ? February the seventh
*SYS: yes

*TEA: tell me the name -s of the fantastic four
*SYS: Mister Fantastic
*TEA: Mister Fantastic
*SYS: The Invisible Woman
*TEA: right
*SYS: The Thing

*TEA: that is right
*SYS: The Human Torch

*TEA: tell me the name -s of the seven kings
*SYS: first Romulus
*TEA: first Romulus
*SYS: second Numa Pompilius
*TEA: second Numa Pompilius
*SYS: third Tullus Hostilius
*TEA: that is good
*SYS: fourth Ancus Marcius
*TEA: right
*SYS: fifth Tarquinius Priscus
*TEA: very good
*SYS: sixth Tarquinius Superbus
*TEA: okay
*SYS: seventh Servius Tullius
