# Supplementary material for: A Cognitive Neural Architecture Able to Learn and Communicate through Natural Language
Source: PLoS One. 2015 Nov 11;10(11):e0140866. doi: 10.1371/journal.pone.0140866 (PMC4641699; doi:10.1371/journal.pone.0140866)
Supplement: S3 Appendix — (PDF) [file pone.0140866.s003.pdf]

Appendix 3 Examples of neural activation patterns

This section describes with examples how the neural activation patterns of the system evolve, how the connection weights are modified during the training stage and how these weight changes make the system able to generalize the response to new sentences. A detailed description of the system architecture is provided in S5 Appendix.

1 First example: categorization

The first example is a simple question-answering task from the categorization dataset. It illustrates what happens to the internal states of the system when it learns to answer questions as "tell me a reptile", and how the changes in the connection weights make the system able to generalize this knowledge to similar questions involving different categories.

1.1 Input phrase acquisition

Fig. 1 illustrates how an input sentence ("the turtle is a reptile" in this example) is acquired by the system and stored in the input phrase buffer. When this sentence is written in the terminal or read from a file, the interface submits its words one by one to the system, using the ascii representation, starting from the word "the". This word is mapped to the *input-word buffer (*IW) through the mechanism described in Sect. 2 of S5 Appendix. The input nodes are fully connected to IW, and the connection weights are initialized randomly. IW is updated using the *winner-take-all* (WTA) rule: the neuron with the highest activation state (winner neuron) is switched to the level one, while all other neurons of IW are switched to zero. The connections from the input nodes to the winner neuron of IW are updated through the *discrete-hebbian-learning* (DHL) rule: if the input node signal is one, the connection weight is saturated to its maximum value (+1), otherwise it is saturated to its minimum value (-1). This ensures that if this word is submitted again to the system, the winner neuron will be the same.

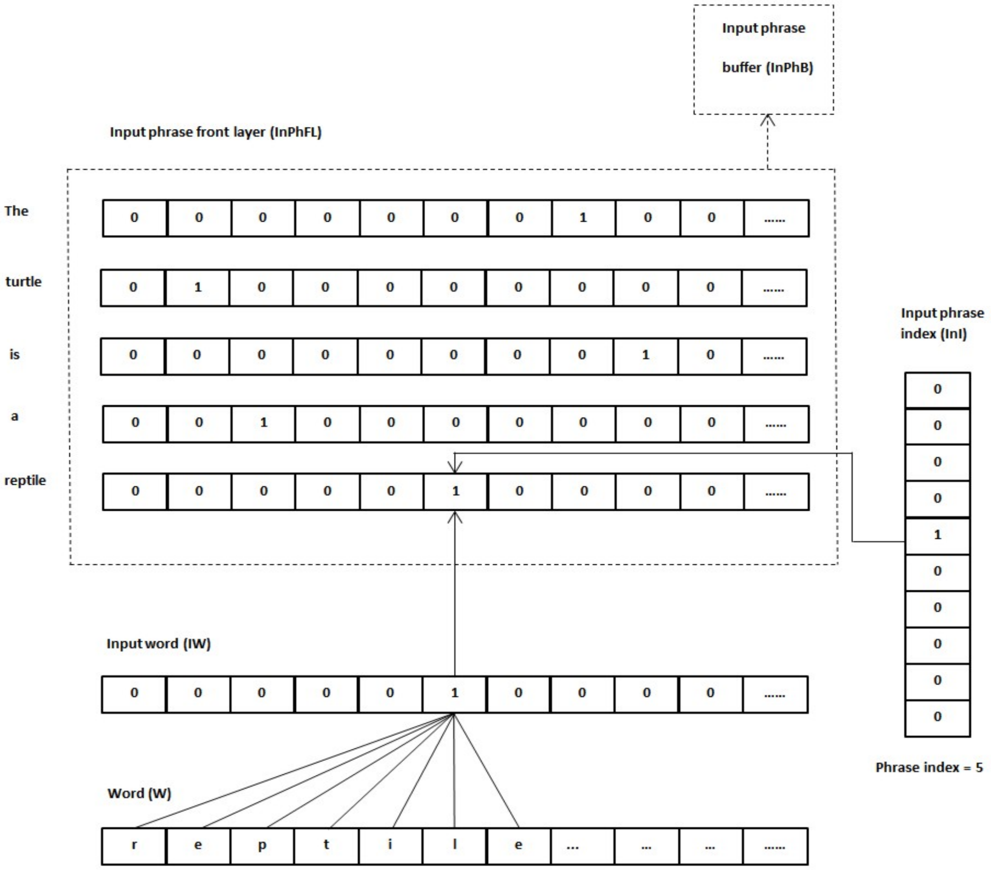

Fig. 1. Input phrase acquisition.

PhI (*phrase index*) is a subnetwork that represents the position of the current word in the phrase: the neuron of PhI corresponding to the position of the word in the phrase is in a high-level state, while all the others are in a low-level state. The words of the input phrase are submitted to the system by loading them, one by one, in the word buffer, and increasing the phrase index from 1 to the number of words in the phrase. The system itself initializes the phrase index at the beginning of a phrase acquisition, and increases it after the acquisition of each word, as discussed in Sect. 9 of S5 Appendix. In this way a couple (word, phrase-index) is mapped to the neuron of InPhFL located in the row i corresponding to the phrase index and in the column j corresponding to the word-mapping neuron index.

This structure is suitable for a broad range of problems in adaptive behavior, not only language understanding. In general, a "word" can be defined as a specific input pattern. The system can associate a key to each word received as input and generate a unique pattern corresponding to the couple (key, word). A "phrase" is set of couples (key, word), temporarily stored in the system. The key

can be any pattern, not necessarily representing an integer number, however in the SSM approach a single neuron or a small number of neurons should be active for any key pattern. In the case of natural language, the "phrase index" is a key that represents the position of each word in a phrase.

The input-phrase front layer is single-connected to the input-phrase buffer (InPhB). The input-phrase buffer is also single-connected to itself (self connection). In this way, it can store all words of a phrase and keep them stored until it is cleared by a flush signal.

1.2 Copy of the input phrase to the working-phrase buffer

After the whole input sentence is acquired, the system executes the action PH_FROM_INPUT, which copies the sentence from the input phrase buffer to the working phrase buffer, as illustrated in Fig. 2. The input-phrase-buffer neurons are connected one by one to the intermediate subnetwork WkPhFL. The action neuron PH_FROM_INPUT activates the gatekeeper neuron WkFlag, which is fully connected to WkPhFL. When the gatekeeper neuron is ON, WkPhFL allows the signal to flow from the input phrase buffer to the working phrase buffer, through the mechanism described in Sect. "*Neural gating mechanisms*".

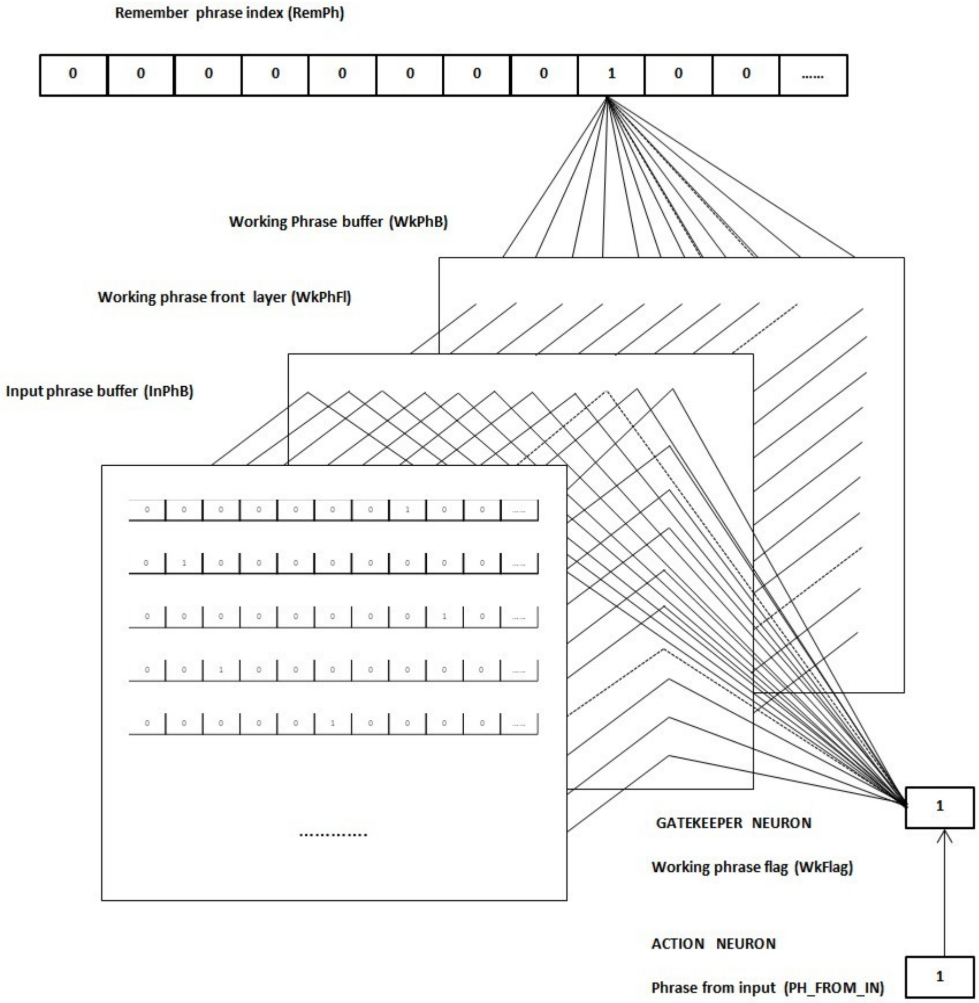

Fig. 2. Copy of the input phrase to the working phrase buffer and memorization of the phrase in long-term memory.

1.3 Memorization of the input phrase in long-term memory

The subnetwork RemPh (remembered phrase) is used as an index for storing and retrieving phrases from long-term memory. RemPh is fully connected to WkPhB by forcing connections. The active neuron of RemPh represents the current phrase index in long-term memory. After the input phrase is copied to WkPhB, the connections from this neuron to WkPhB are updated through the DHL rule. In this way, if this neuron is switched ON again, it will retrieve the memorized phrase by forcing the activation states of WkPhB.

1.4 Extraction of a word-group from the working-phrase buffer

Fig. 3 shows how a word is extracted from the working phrase buffer. PhI (phrase index) represents the index of the word in the phrase. Each neuron of the intermediate subnetwork WkWfI performs a logical AND between the corresponding neuron of WkPhB and that of PhI. In this way, the row of WkPhB corresponding to the phrase index is copied to the subnetwork that represents the current word, CW.

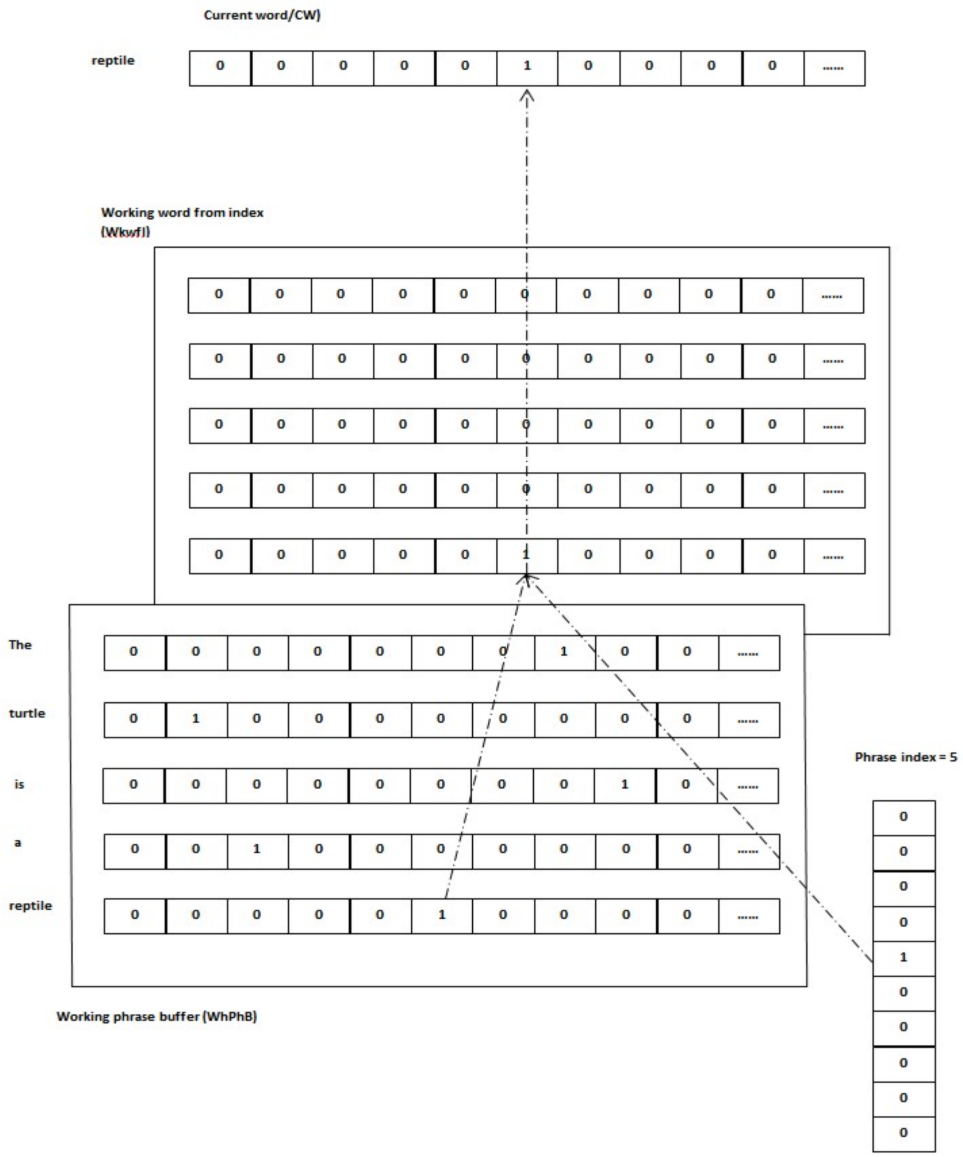

Fig. 3. Extraction of the current word from the working phrase buffer.

The current word can be extracted from CW and copied to the word-group buffer through the procedure illustrated in Fig. 4, which is controlled by the gatekeeper neuron GetFlag. When GetFlag is ON, the intermediate subnetwork WGCW (word-group current word) allows the flow of signal from CW to WGFL (word-group front layer), which operates a logical AND between this signal and the word-group index WGI. In this way, the current word is copied to the row of WGFL that corresponds to the word group index.

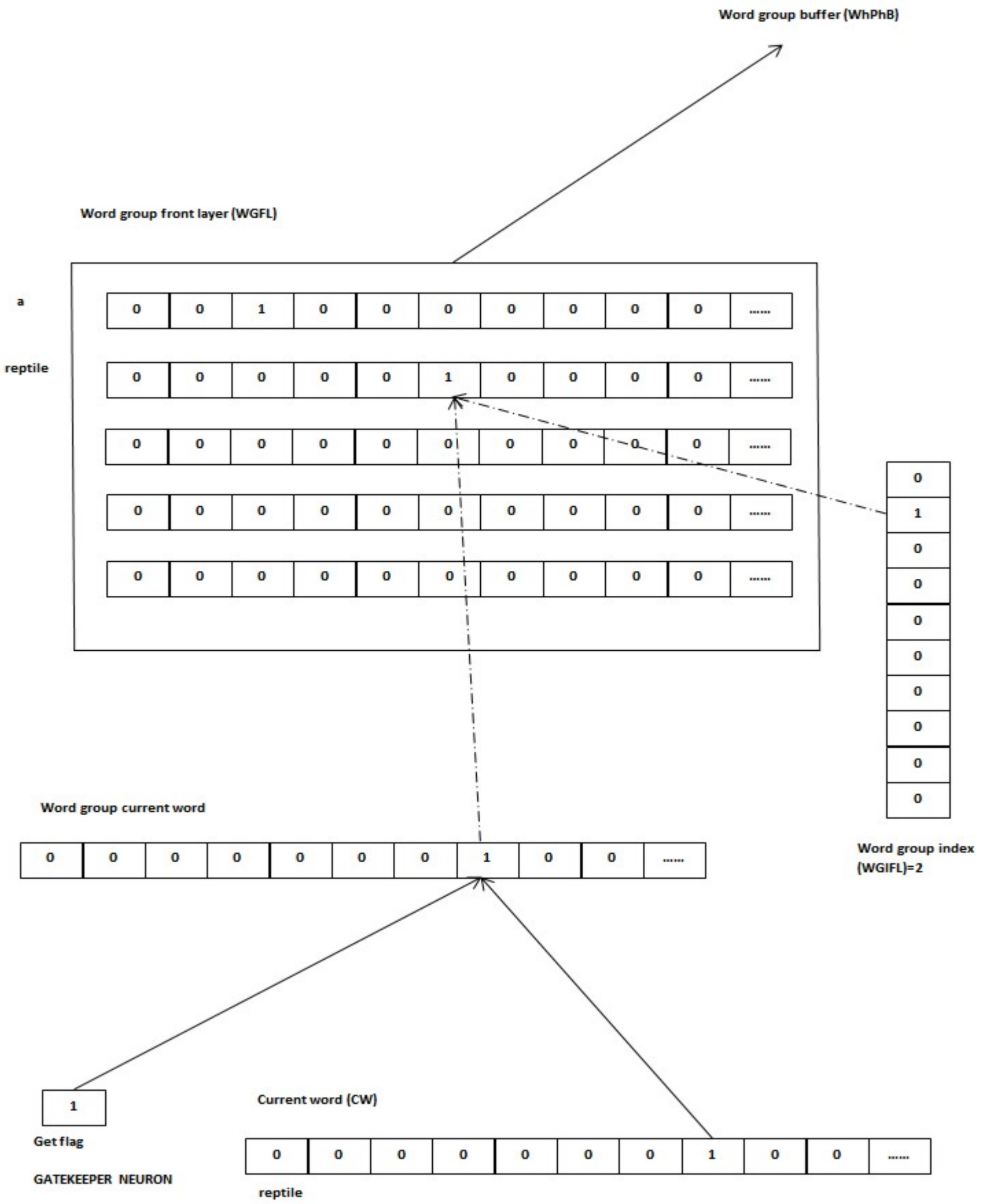

Fig. 4. Copy of the current word to the word group buffer.

1.5 Memorization and retrieval of the association between a word group and a phrase

The group of words in WGB can be used as a cue to retrieve a phrase from long-term memory. Fig. 5 shows how the association between a group of words and the whole phrase is stored in long-term memory. The word group buffer is fully connected to the subnetwork RemPhfWG (remembered-phrase from word group), which is fully connected to RemPh by forcing connections. RemPhfWG is updated through the WTA rule. The connections from WGB to the winner neuron and the connections from this neuron to RemPh are updated through the DHL rule.

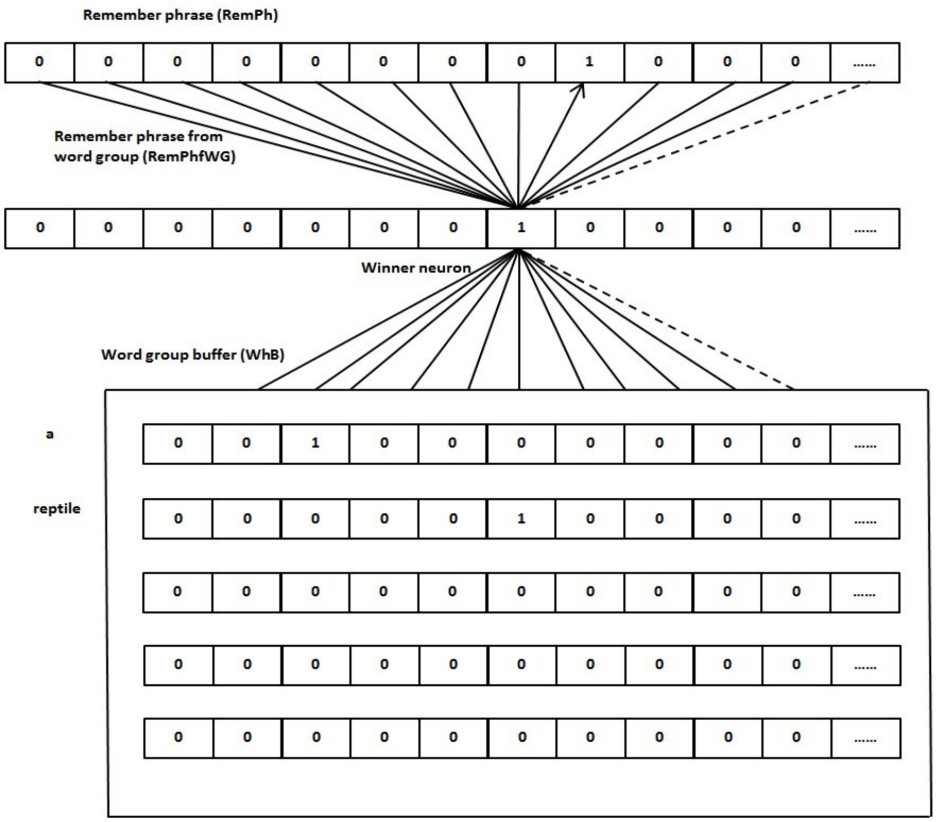

Fig. 5. Memorization and retrieval of the association between a word group and a phrase.

The association between the word group in WGB and the phrase in WkPhB is memorized in long-term memory using the architecture shown in Fig. 5. The word group buffer is fully connected to

RemPhfWG, which is fully connected to RemPh by forcing connections. RemPhfWG is updated through the *winner-take-all* (WTA) rule. The weights of the connections from WGB to the winner neuron and the weights of the connections form this neuron to RemPh are updated through the DHL rule. In this way, the association between the current content of WGB and the current content of RemPh is permanently memorized by the system. During the retrieval process, the word group in WGB is sent as input to RemPhfWG. The neurons having connection weights matching the word group will have the highest activation state, and a single winner is selected among them through the WTA rule. The winner neuron will retrieve the phrase associated to the input word group by using its forcing output connections to set the activation state of WkPhB.

1.6 Exploration

The system is trained to respond to the input sentences through an exploration/reward procedure. Following our example, suppose that the human interlocutor submits the question-like imperative sentence

tell me a reptile

During the exploration phase, the system performs partially random action sequences.

The basic action sequence is that described in Sect. "*Global organization of the model*":

- *W_FROM_WK*
- *NEXT_W* (N_1 times)
- *FLUSH_WG*
- *GET_W, NEXT_W* (N_2 times)
- *RETR_AS*

with N_1, N_2 random integer numbers. The action neuron NEXT_W activates the gatekeeper neuron NextPhIFlag, which triggers an increase of the phrase index PhI, as described in Sect. 9 of S5 Appendix. The action neuron GET_W activates the gatekeeper neuron GetFlag, which controls the copy of the current word from the working phrase to the word group buffer, as described previously. The action neuron RETR_AS activates the gatekeeper neuron RetrAs, which controls the retrieval of a phrase from long-term memory using the word group as a cue as discussed in the above paragraphs.

The whole sequence is repeated, using different random integer values for N_1, N_2, until it produces the target output. In our example this can occur, for instance, with $N_1=2$ and $N_2=2$. In fact, in this case the system extracts the word group “a reptile” from the input phrase “tell me a reptile”. The RETR_AS action uses this word group as a cue, and can eventually retrieve the phrase “the turtle is a reptile” from long-term memory. The basic action sequence is repeated on the new working phrase, and the system produces the target output “turtle” if $N_1=1$ and $N_2=1$.

The state-action sequences are memorized through the mechanism represented in Fig. 6. The state-action index StActI is initialized to one at the beginning of each sequence, and it is increased every time the system produces a new action. The neurons of StActI are connected one by one to the corresponding neurons of StActMem (state-action memory), which is fully connected to all subnetworks that represent the internal state of the system state (as defined in Sect. “*Global organization of the model*”) and to the action neurons. The output connections of StActMem are updated through the DHL rule. In this way, StActMem can retrieve the state-action sequence by forcing the activation state of the neurons connected to its output connections.

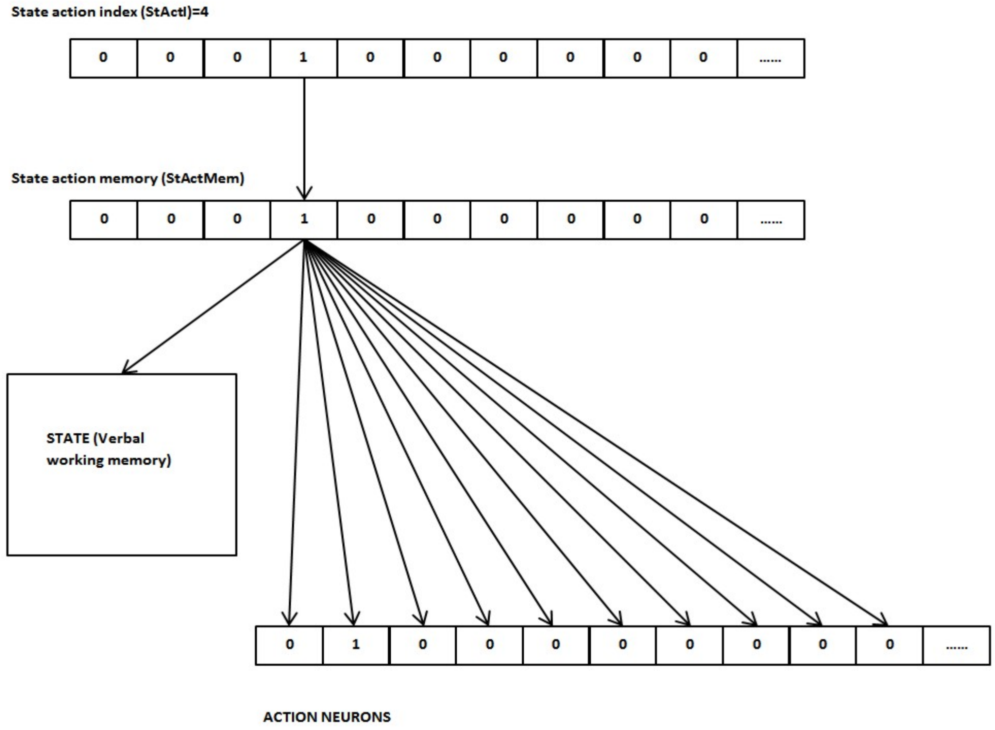

Fig. 6. Memorization and retrieval of a state-action sequence.

1.7 Reward

When the exploration phase leads to the target output, the system is set to the reward operating mode. The memorized state-action sequence is retrieved, as described in the previous paragraphs.

The association between each state of the sequence and the corresponding action is memorized through the state-action association subnetwork ElActfSt, which has input connections fully connected to the system state and output connections fully connected to the action neurons, as illustrated in Fig. 7. In the reward operating mode, ElActfSt is updated through the WTA rule, and both the connections from the verbal working memory to the winner neuron of ElActfSt and the connections from this neuron to the action neurons are updated through the DHL rule.

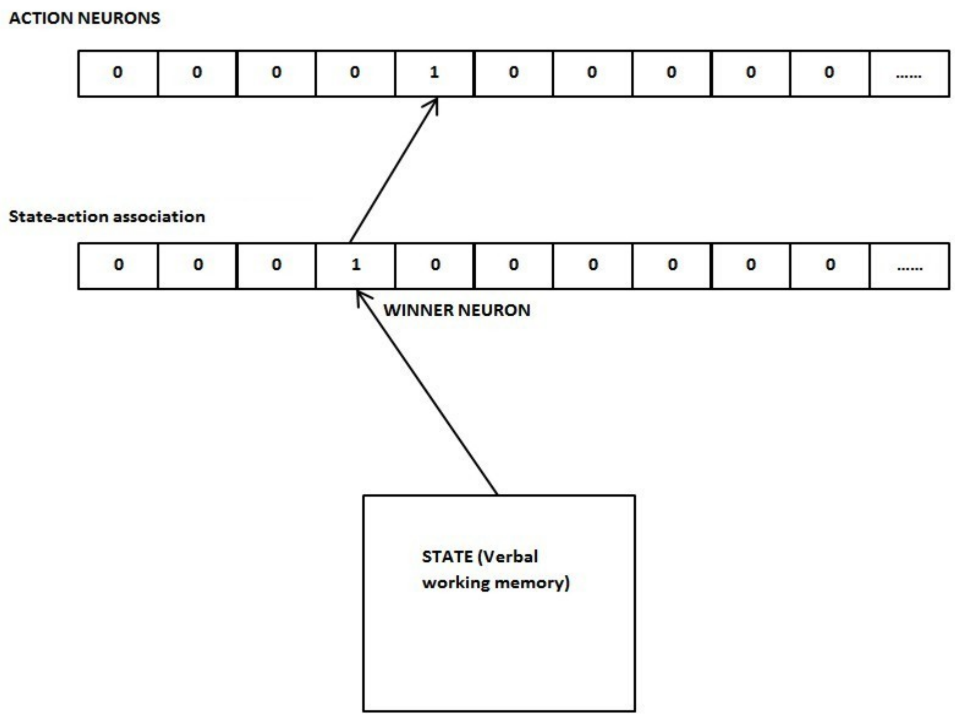

Fig. 7. State-action association.

1.8 Exploitation and generalization

Following our example, suppose that the human interlocutor types the sentence

tell me a mammal

and that after the acquisition the system is set to the exploitation operating mode.

The state-action association subnetwork ElActfSt receives its input from the system state, and it is updated through the k-WTA rule: the k neurons that have the highest activation state are set to one, while all other neurons are set to zero. Those neurons send their output to the action neurons, which are updated through the WTA rule: the action neuron with the highest activation state (which represents the action with the highest score) is set to one, while all other action neurons are set to zero.

Although the input phrase is new for the system, it is similar to the one used for training ("tell me a reptile"). Therefore, at each step of the exploitation phase, the neurons of the central executive with the highest activation state will be those that have been rewarded in the training example, and consequently the action sequence will be the same, i.e.

- *W_FROM_WK*
- *NEXT_W* (2 times)
- *FLUSH_WG*
- *GET_W, NEXT_W* (2 times)
- *RETR_AS*
- *W_FROM_WK*
- *NEXT_W* (1 time)
- *FLUSH_WG*
- *GET_W, NEXT_W* (1 time)

Through such sequence, the system will extract the word group "a mammal" from the working phrase, retrieve a phrase as "the dog is a mammal" from long-term memory, extract the word "dog" and send it to the output.

Fig.s 8 and 9 show a comparison of some STM components in a step of the training stage and in the corresponding step of the test stage, respectively. Those figures also show the relevant connections of the STM components to a winner neuron of the state-action association system. In the training step, the connections from the STM neurons that are "on" to the winner neuron of the state-action association system and the connection from this neuron to the action neuron are saturated to their maximum value by the DHL rule.

It can be observed from the two figures that many of the neurons that were “on” in the training step are also “on” in the corresponding test step. Therefore, the same neuron of the state-action association system will have a high input signal, and thus it will be one of the winner neurons in the test stage too.

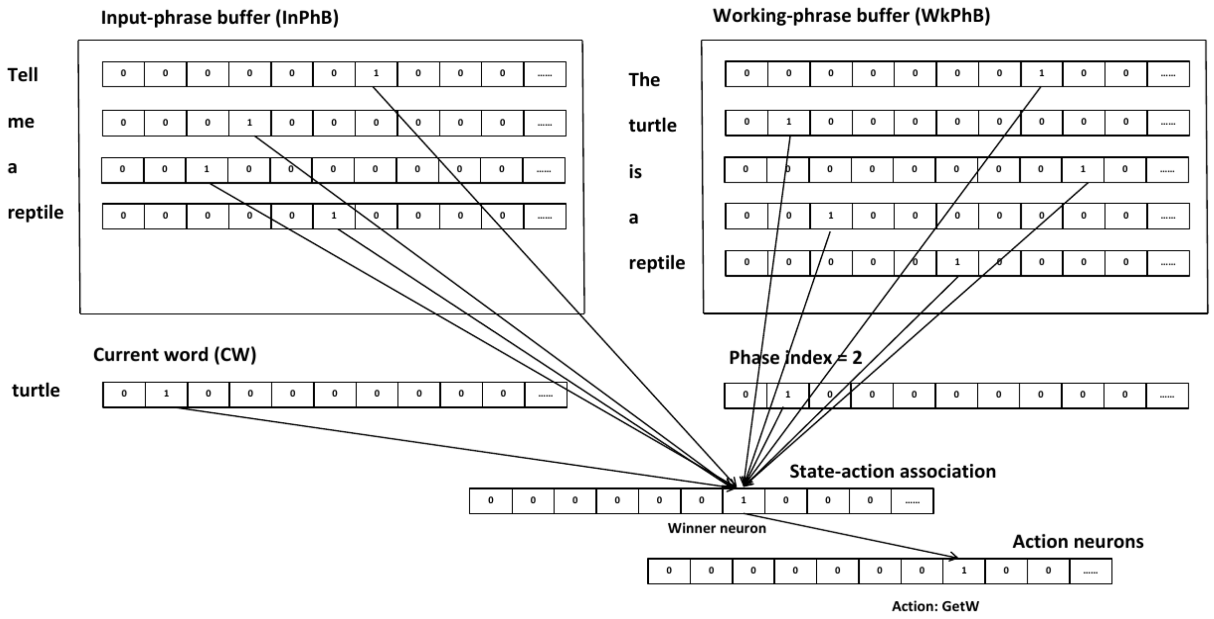

Fig. 8. Neural activations of some relevant STM components in a step of the training stage, and connections from these components to the state-action association system. Only the connections that are modified by the DHL rule are shown.

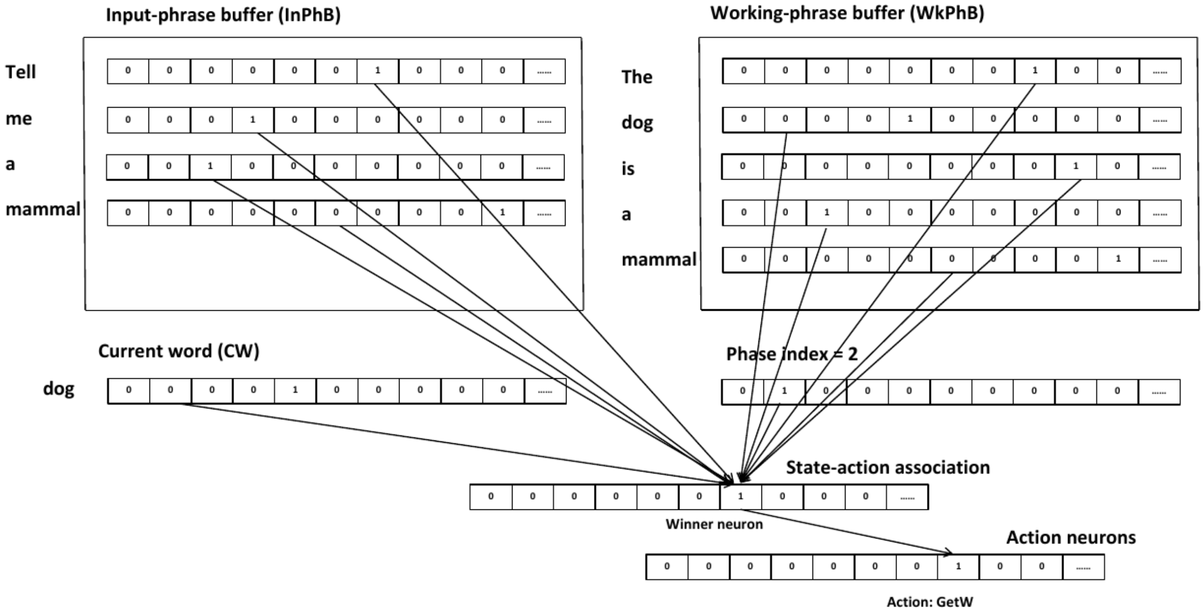

Fig. 9. Neural activations of some relevant STM components in a step of the test stage, and connections from these components to the state-action association system. Only the connections that have been modified by the DHL rule in the corresponding step of the previous training stage are shown.

2 Second example: adding a small number to a sequence of digits

The example in this section illustrates how the internal states of the system evolve during a more complex cognitive task, which involves simple addition to a sequence of digits, as described in Sect. "*The mental action sequence*". In this example we explain how the system can generalize the acquired knowledge to new numbers, and how the goal stack and the comparison structure are able to facilitate retrieval of the correct phrases from LTM.

As in the experiment with a human subject, this example assumes that the system has memorized additions with small numbers, so that the cognitive load for a single addition is small. To ensure this condition, the following sentences are submitted to the system before training:

one plus one equals two

two plus one equals three

….

nine plus one equals ten

and analogous sentences with “plus two” and “plus three”. Then the system is trained with the sentence

add the number two to the digits six three nine four .

After the acquisition of the phrase “add the number two”, the teacher suggests the system to focus the attention on the word “two” and to push this word and the phrase onto the stack.

.wg two *# focus the attention on this word*

.push_goal *# push the working phrase and the word group on the goal stack*

The mechanism used to transfer the working phrase and the word group to the goal stack is analogous to that described in Sect. 1.2 of this appendix. Note that only the first level of the stack structure is used in this example. The whole structure of the stack is described in Sect. 14 of S5 Appendix.

Subsequently, the phrase “to the digits six three nine four” is acquired, and the teacher suggests the system to focus the attention on the word “six”, to retrieve the target phrase “six plus two equals eight”, to extract the word “eight” and to send this word to the output:

.wg six *# focus the attention on this word*

.ph six plus two equals eight *# retrieve this phrase*

.wg eight *# extract this word*

.prw *# partial output and reward*

All these actions are performed in an analogous way as described in the previous section. After the first output, the same procedure is applied to the second digit of the sentence, and so on.

Fig. 10 shows the state of some relevant STM components when the system retrieves the phrase “six plus two equals eight”. Fig. 11 shows how the system updates the part of the comparison structure that compares the working phrase with the goal stack. The intermediate subnetwork performs a logical AND between the working-phrase buffer and the goal-word-group buffer. In this way, the comparison vector signals that the third word of the working phrase is equal to the first word of the goal word group.

In the test stage, the sentence “add the number three to the sequence seven eight two five” is submitted to the system. As this sentence is similar to the one that was used for training, in the exploitation stage the system will perform the same set of actions; after pushing the phrase “add the number three” onto the stack, it will extract the word “seven” from the working phrase, use this word

as cue to retrieve the sentence “seven plus three equals ten” from LTM, extract the word “ten”, send this word to the output, and so on with the other digits of the sequence.

Fig. 12 illustrates the state of the relevant STM components when the system retrieves the phrase “seven plus three equals ten”. It can be observed that there is a significant correspondence between the active neurons of Fig. 12 and those of Fig. 10. This implies that the STM state in this step of the test stage is close to the corresponding step of the training stage, in terms of distance in the vector space that represents the STM states. This similarity makes the state-action association system able to generalize the knowledge acquired in the training stage: the k neurons that won the k-winner-take-all competition in the training stage will have a high input signal in the corresponding step of the test stage, and therefore they will activate the same action neuron.

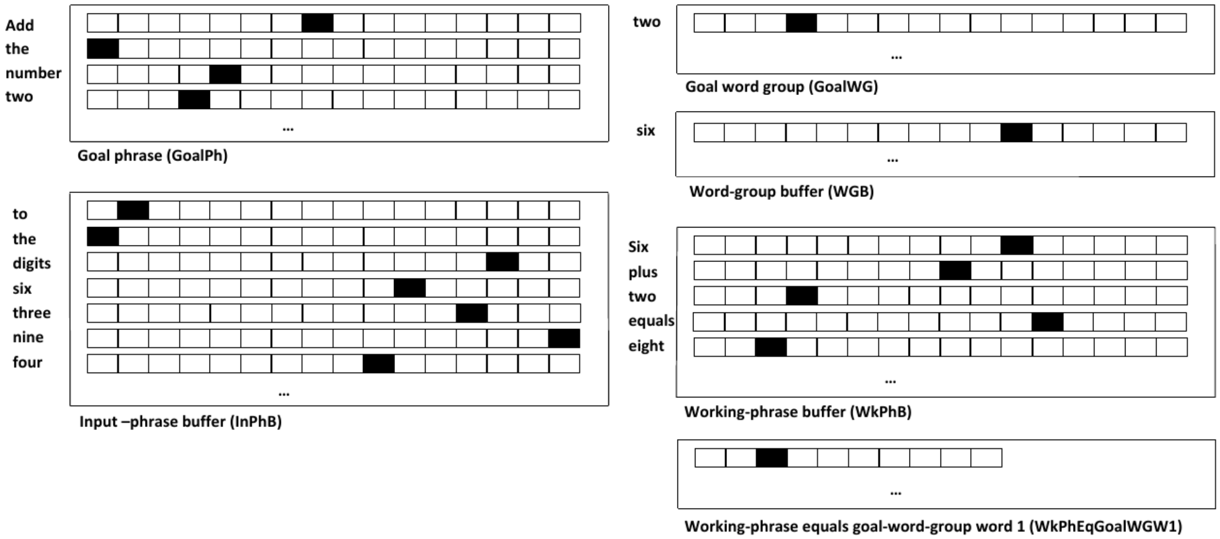

Fig. 10. Neural activations of some relevant STM components in a step of the training stage.

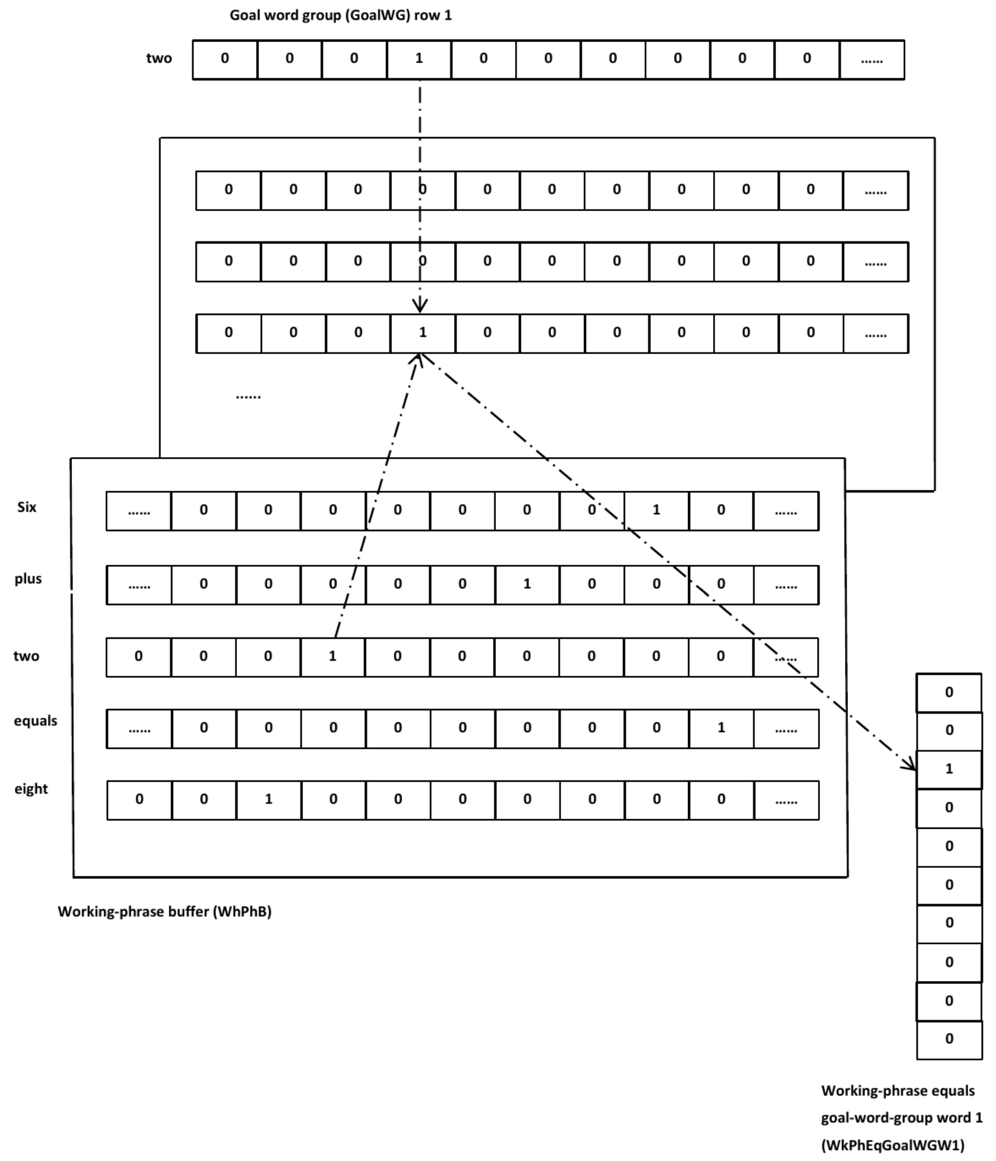

Fig. 11. Comparison between the working phrase and the goal stack. The intermediate subnetwork performs a logical AND between the working-phrase buffer and the goal-word-group buffer, and sends the output to the corresponding part of the comparison structure.

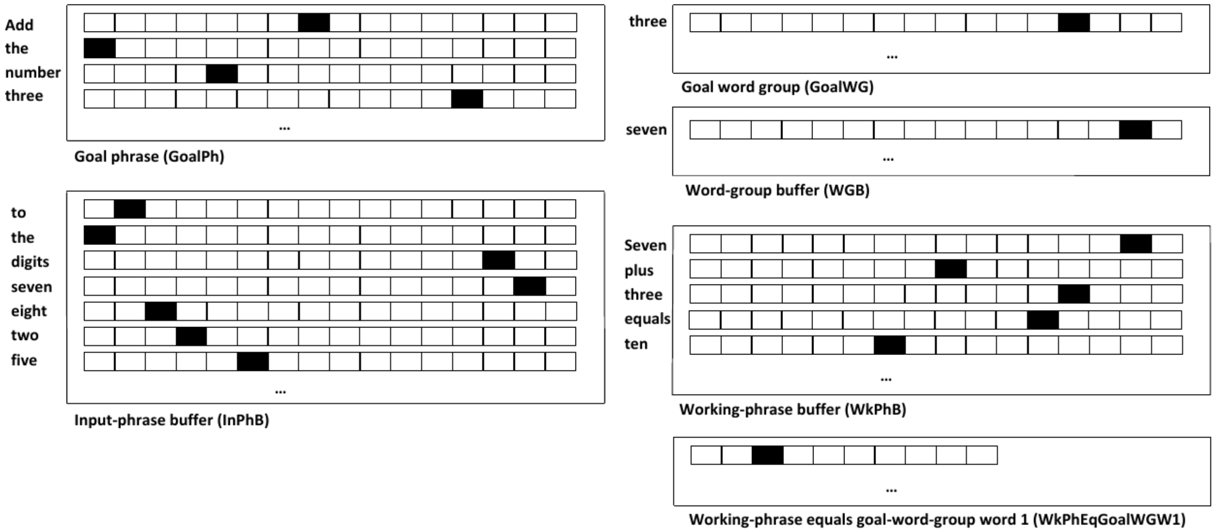

Fig. 12. Neural activations of some relevant STM components in a step of the test stage.
