# Supplementary material for: A Cognitive Neural Architecture Able to Learn and Communicate through Natural Language
Source: PLoS One. 2015 Nov 11;10(11):e0140866. doi: 10.1371/journal.pone.0140866 (PMC4641699; doi:10.1371/journal.pone.0140866)
Supplement: S4 Appendix — (PDF) [file pone.0140866.s004.pdf]

Appendix 4 Mathematical properties of the state-action association system

The heart of the ANNABELL model is the state-action association system, which is responsible for all decision processes, as described in Sect. "*Global organization of the model*". This system is implemented as a neural network (state-action association neural network, abbreviated as SAANN) with input connections fully connected to all subnetworks of the short-term memory (STM), which represents the internal state of the system, and output connections fully connected to the set of mental action neurons. Therefore, the SAANN receives as input the internal state and yields as output a mental action. The input and output connections of this system have learnable weights, which are updated through a discrete version of the Hebbian learning rule (DHL rule). Furthermore, the activation states of the SAANN are updated through a variant of the k-winner-take-all rule, while those of the action neurons are updated through the (one-) winner-take-all rule.

In this section, we describe the update rules in more details and we prove that our model of the state-action association system is equivalent to a k-nearest-neighbor (k-NN) classifier with a proper definition of the distance in the input space. For large enough training sets, the k-NN algorithm is guaranteed to yield an error rate no worse than twice the Bayes error rate, which is the minimum achievable error rate given the distribution of the data [1].

The discrete-Hebbian-learning (DHL) rule used in our model is an extreme simplification compared with other models more focused on biological realism. The same type of simplification is often used in neural models of memory based on the Hopfield recurrent neural networks [2].

Nessler et al. [3] have proven that a more realistic model of Hebbian learning, in combination with a sparse neural code, can learn to infer optimal Bayesian decisions for arbitrarily complex probability distributions. However, a more realistic implementation of the Hebbian learning rule, with small updates of the connection weights, would require very large computational resources for training and evaluating our model on large datasets, and real time interaction with the system would not be possible. O'Reilly [4] have shown that the k-winner-take-all rule is biologically justified.

Other simplifications are used in our model:

- *stability condition*: the proof that the state-action association system is equivalent to a k-NN classifier assumes that the STM can be partitioned into *M* subnetworks each having a fixed number of neurons active at a time. The weight saturation value W_{max}, used by the DHL rule, is

assumed to be the same for all connections of the same subnetwork. A particular case is when the whole STM has a fixed number of neurons active at a time, and $W_{\max}$ has the same value for all connections from the STM to the SAANN. In the subnetworks that represent words or phrases, the stability condition is ensured by using default neurons, which represent the null word. In the subnetworks used for word comparison, such property is fulfilled by representing the two conditions, equal/not-equal word, using two complementary neurons instead of one.

- During the training stage, the SAANN is updated through the new-winner-take-all rule: a previously unused neuron is set to the high-level activation state ("on"), while all other neurons are set to the low-level activation state ("off").

Those two simplifications are used only for ensuring validity of the k-NN equivalence theorem, so that the statistical properties of the model are contextualized in a well-known theoretical framework, and good convergence properties of the error rate are guaranteed.

It is worth to mention that many biologically inspired neural models of language use the standard backpropagation learning algorithm, even though it does not have a biological justification, because it ensures error minimization. In contrast, our model is based on the same learning principle that is responsible for synaptic plasticity in biological neural networks.

Let A_m and $W_{\max,m}$ be the number of active neurons and the weight saturation value of the mth subnetwork, respectively. The stability condition ensures that A_m is constant. Let s_{mj} be the activation state (0 or 1) of the jth neuron of the mth subnetwork. The sum and the square sum of s_{mj} weighted with $W_{\max,m}$ are the following:

$$\sum_{m=1}^{M} W_{\max,m} \sum_{j=1}^{N_m} s_{mj} \quad \text{and} \quad \sum_{m=1}^{M} W_{\max,m} \sum_{j=1}^{N_m} s_{mj}^2 \tag{1}$$

where N_m is the number of neurons of the mth subnetwork. The stability condition implies that, for all values of m,

$$\sum_{j=1}^{N_m} s_{mj} = \sum_{j=1}^{N_m} s_{mj}^2 = A_m \tag{2}$$

therefore the following *normalization conditions* can be derived for the weighted sum and for the weighted square sum of the signal:

$$\sum_{m=1}^{M} W_{\max,m} \sum_{j=1}^{N_m} s_{mj} = U_1 \quad \text{and} \quad \sum_{m=1}^{M} W_{\max,m} \sum_{j=1}^{N_m} s_{mj}^2 = U_2 \tag{3}$$

where

$$U_1 = U_2 = \sum_{m=1}^{M} W_{\max,m} A_m \tag{4}$$

are constants. It is worth to point out that these two normalization conditions are sufficient for the validity of the k-NN equivalence theorem, which we will prove below, even if the stability condition is not satisfied.

The weighted distance between two states S_1 and S_2 of the STM can be defined as:

$$d(S_1, S_2) = \sum_{m=1}^{M} W_{\max,m} \sum_{j=1}^{N_m} (s_{1mj} - s_{2mj})^2 = 2U_2 - 2\sum_{m=1}^{M} W_{\max,m} \sum_{j=1}^{N_m} s_{1mj} s_{2mj} \tag{5}$$

where we used the second normalization condition of Eq. 3.

Let N_A be the number of mental action neurons, i.e. the number of possible actions that can be triggered by the state-action association system. A mental action can be represented by an integer value:

$$a = 1, \ldots, N_A \tag{6}$$

A state-action sequence, starting with a state S^{α}_1 and ending in a state $S^{\alpha}_{T\alpha}$ will be called an epoch:

$$(S^{\alpha}_1, a^{\alpha}_1), \ldots, (S^{\alpha}_t, a^{\alpha}_t), \ldots, (S^{\alpha}_{T\alpha}, a^{\alpha}_{T\alpha}) \quad . \tag{7}$$

The index α represents the epoch, while the index t represents the time step in the epoch:

$$t = 1, \ldots, T_{\alpha} \quad . \tag{8}$$

The number of time steps in all epochs is limited: $T_{\alpha} \leq T_{\max}$. An epoch can receive a reward depending only on its final state. In the reward phase, the state-action memory retrieves the whole state-action sequence, and the SAANN is trained using the state S^{α}_t as input and the corresponding action a^{α}_t as target output. At each time step t of the sequence, the SAANN is updated using the new-winner-take-all rule: a previously unused neuron i is set to the "on" state, while all other neurons are set to the "off" state. The connections from the STM to the winner neuron are updated through the DHL rule:

$$w_{imj} = \begin{cases} +W_{\max,m} & \text{for } s^{\alpha}_{tmj} = 1 \\ -W_{\max,m} & \text{for } s^{\alpha}_{tmj} = 0 \end{cases} \tag{9}$$

where the two indexes m and j refer to the jth neuron of the mth subnetwork of the STM, s^{α}_{tmj} is the activation state of this neuron (0 or 1) at the epoch α and time step t, w_{imj} is the weight of the

connection to the ith neuron of the SAANN (i.e. the winner neuron) and $W_{max,m}$ is the weight-saturation absolute value for the subnetwork m. These two equations can also be written as:

$$w_{imj} = W_{\max,m}(2 s^{\alpha}_{tmj} - 1) \tag{10}$$

The connections from the winner neuron of the SAANN to the action neurons are also updated through the DHL rule:

$$w_{li} = \begin{cases} +1 & \text{for } l = a^{\alpha}_{t} \\ -1 & \text{for } l \neq a^{\alpha}_{t} \end{cases} \tag{11}$$

where the index l refers to an action neuron, w_{li} is the weight of the connection from the winner neuron of the SAANN to this action neuron and a^{α}_{t} is the target action.

During the exploitation phase, in general the internal states will be different from those used in the training phase. Let S^{test} be a generic internal state of the system in the exploitation phase. The total input signal to each neuron of the SAANN is:

$$y_i = \sum_{m=1}^{M} \sum_{j=1}^{N_m} w_{imj} s^{\text{test}}_{mj} \tag{12}$$

where the bias signal is assumed to be null. From Eq. 10 for w_{imj} it follows that

$$y_i = \sum_{m=1}^{M} [W_{\max,m} \sum_{j=1}^{N_m} (2 s^{\alpha}_{tmj} - 1) s^{\text{test}}_{mj}] = 2 \sum_{m=1}^{M} W_{\max,m} \sum_{j=1}^{N_m} s^{\alpha}_{tmj} s^{\text{test}}_{mj} - U_1 \tag{13}$$

where we used the first normalization condition of Eq. 3, and using Eq. 5 for the weighted distance:

$$y_i = 2U_2 - U_1 - d(S^{\alpha}_{t}, S^{\text{test}}) \tag{14}$$

In the exploitation phase, the SAANN is updated through the k-winner-take-all rule: the k neurons with the highest activation state are set "on", while all the others are set "off".

Since the activation function $f(y_i)$ is an increasing function of the input signal y_i, from Eq. 14 it follows that the neurons with the highest activation state y_i are those with the smallest value of $d(S^{\alpha}_{t}, S^{\text{test}})$. Therefore, the k neurons with the highest activations are those that correspond to the training internal states that have the smallest weighted distance from the current (test) internal state, i.e. to the k nearest neighbors with such metric.

Each "used" neurons of the SAANN, i.e. each neuron that was classified as a winner neuron during a reward phase, is connected with a positive-weight connection ($w_{li} = +1$) to one and only one action neuron, while it is connected to all other action neurons by negative-weight connections ($w_{li} = -1$).

We can therefore partition the used neurons of the SAANN in classes, based on the action that they "suggest".

The input signal to each action neuron is equal to the weighted sum of the input from the *k* winner neurons. Since the output of the winner neurons is 1, and the weights are $w_{li} = \pm 1$, the input signal is equal to the number of winner neurons that "suggest" that action as the best action, minus the number of those that do not. The action neuron with the highest input signal is the one that is "suggested" as the best action by the greatest number of winner neurons. The actions neurons are updated by the (one) winner-take-all rule, therefore this neuron will be set "on", while all the other action neurons will be set "off". This is equivalent to a k-NN classification. In fact, in k-NN classification an entry is assigned to the class most common among its *k* nearest neighbors.

References

1. Cover TM, Hart PE (1967) Nearest neighbor pattern classification. *IEEE Transactions on Information Theory* 13(1): 21–27 (1967)
2. Haykin S (1998) *Neural Networks: A Comprehensive Foundation* (Prentice Hall PTR Upper Saddle River, NJ, USA, 2nd edition).
3. Nessler B, Pfeiffer M, Maass W (2009) Hebbian learning of Bayes optimal decisions. Proceedings of the 21th Conference on Advances in Neural Information Processing Systems; December 2008. Vancouver, Canada: NIPS 2008.
4. O'Reilly RC, Munakata Y (2000) *Computational Explorations in Cognitive Neuroscience: Understanding the Mind by Simulating the Brain* (MIT Press, Cambridge, MA).
