# Supplementary material for: A Cognitive Neural Architecture Able to Learn and Communicate through Natural Language
Source: PLoS One. 2015 Nov 11;10(11):e0140866. doi: 10.1371/journal.pone.0140866 (PMC4641699; doi:10.1371/journal.pone.0140866)
Supplement: S5 Appendix — (PDF) [file pone.0140866.s005.pdf]

Appendix 5 The ANNABELL system architecture

(Version 2.0.9)

Table of Contents

Appendix 5 The ANNABELL system architecture....1
1 Type of connections and notations....2
2 Input phrase acquisition....4
3 Copy of the input phrase to the working-phrase buffer....6
4 Extraction of a word from the working-phrase buffer....7
5 Copy of a word from the current-word buffer to the word-group buffer....8
6 Copy of the word group to the output buffer....9
7 Memorization of a phrase....10
8 Memorization and retrieval of the association between a word group and a phrase....11
9 Phrase index and word-group index update....12
10 Memorized phrase and remembered phrase index update....13
11 Equal-words vectors....14
12 Previous working phrase, previous word-group and previous equal-word vectors....17
13 Set and retrieve the starting phrase of a context....20
14 The goal stack....22
15 Goal equal-words vectors....25
16 Action and gatekeeper neurons....26
17 Memorization and retrieval of a state-action sequence....29
18 State-Action association....30
19 Operating-modes subnetworks....31
19.1 Acquisition....32
19.2 Association....33
19.3 Exploration....35
19.4 Reward....41
19.5 Exploitation....42

1 Type of connections and notations

Figure 1 represents the notations used in the following diagrams. Plain (one-dimensional) SSMs are represented by rectangles with a solid line. Generally, all neurons in the same SSM have the same bias, which is reported on the bottom-right corner of the rectangle.

In two-dimensional SSMs (2D SSMs), the neurons are arranged on a two-dimensional array. 2D SSMs are represented by rectangles with a dashed line.

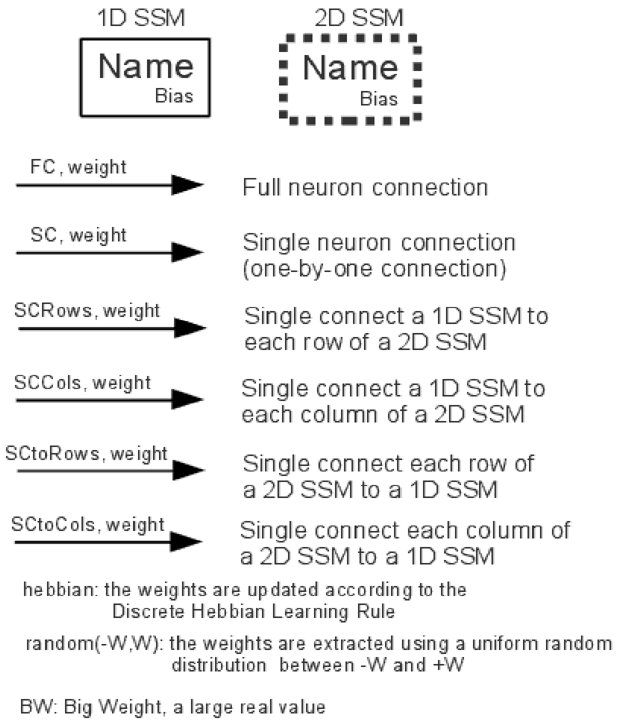

Figure 1. Types of connections and notations.

SSMs can be connected to each other in the following ways:

- full connection (FC): all neurons of the output SSM are connected to all neurons of the intput SSM;
- single connection (SC): each neuron of the output SSM is connected to the corresponding neuron of the input SSM; the input and the output SSMs must have the same size;
- Single connect the rows (SCRows): the output SSM is a 2D SSM, with each row single-connected to the input SSM; The size of the input SSM must be equal to the number of columns of the output SSM;
- Single connect the columns (SCCols): the output SSM is a 2D SSM, with each column single-connected to the input SSM; The size of the input SSM must be equal to the number of rows of the output SSM;
- Single connect to the rows (SCtoRows): the input SSM is a 2D SSM. The output SSM is single-connected to each row of the input SSM; The size of the output SSM must be equal to the number of columns of the input SSM;
- Single connect to the columns (SCtoCols): the input SSM is a 2D SSM. The output SSM is single-connected to each column of the input SSM; The size of the output SSM must be equal to the number of rows of the input SSM;

In case of fixed-weight links, the weight is indicated next to the type of connection. Variable-weight links, which are updated through the discrete Hebbian learning rule (DHL rule), are indicated by the word "hebbian".

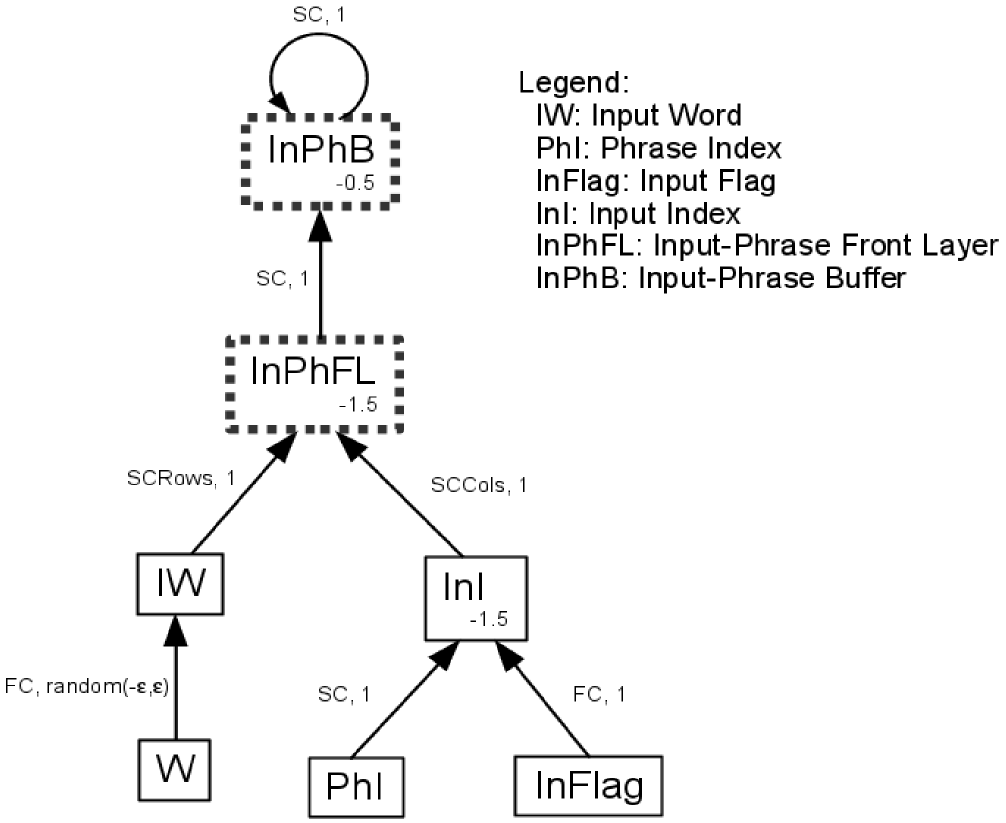

Figure 2. storage of a word in the Input Phrase Buffer.

2 Input phrase acquisition

Figure 2 represents a schematic diagram of the architecture used for input-phrase acquisition. When a sentence is written in the terminal or read from a file, the interface submits its words one by one to the system. Each word is converted to a binary pattern, based on its ascii representation, and submitted to the system input W. The input nodes are fully connected to the input-word buffer (IW), and the link weights are initialized randomly. IW is updated using the *winner-take-all* (WTA) rule: the neuron with the highest activation state (winner neuron) is switched to the level one, while all other neurons of IW are switched to zero. The links from the input nodes to the winner neuron of IW are updated through the *discrete-hebbian-learning* (DHL) rule: if the input node signal is one, the link weight is saturated to

its maximum value (+1), otherwise it is saturated to its minimum value (-1). This ensures that if this word is submitted again to the system, the winner neuron will be the same.

PhI (*phrase index*) is a SSM that represents the position of the current word in the phrase: the neuron of PhI corresponding to the position of the word in the phrase is in a high-level state, while all the others are in a low-level state. The words of the input phrase are submitted to the system by loading them, one by one, in the word buffer, and increasing the phrase index from 1 to the number of words in the phrase. The system itself initializes the phrase index at the beginning of a phrase acquisition, and increases it after the acquisition of each word, as it will be discussed in Sect. 9.

This structure is suitable for a broad range of problems in adaptive behavior, not only language understanding. In general, a "word" can be defined as a specific input pattern. The system can associate a key to each word received as input and generate a unique pattern corresponding to the couple (key, word). A "phrase" is set of couples (key, word), temporarily stored in the system. The key can be any pattern, not necessarily representing an integer number, however in the SSM approach a single neuron or a small number of neurons should be active for any key pattern. In the case of natural language, the "phrase index" is a key that represents the position of each word in a phrase.

The SSM InI (*input index*) in Fig. 2 is single-connected to the phrase index PhI and fully connected to the gatekeeper neuron InFlag (Input Flag). The neuron InFlag is used to control the acquisition: when it is on, a word can be acquired in the input phrase buffer. The bias of InI is set in such a way that if InFlag is on, then InI is simply a copy of the phrase index PhI, otherwise it is blank (all neurons off).

InPhFL (input-phrase front layer) is a two-dimensional SSM (2DSSM) having a number of rows equal to the size of InI and a number of columns equal to the size of the input word buffer IW. Each row of InPhFL is single-connected to IW, while each column of InPhFL is single-connected to InI. Therefore, the neuron (i, j) in row i, column j of InPhFL, is connected to neuron i of InI and to neuron j of IW. The link weights and the bias of InPhFL are set in such a way that the neuron (i, j) will be on only if both input neurons are on. In this way a couple (word, phrase-index) is mapped to the neuron of InPhFL located in the row i corresponding to the phrase index and in the column j corresponding to the word-mapping neuron index.

In biological brains there are no well-ordered subnetworks as the two-dimensional SSM described above. However, they can be considered as a computationally effective model of biologically plausible structures. In fact, consider a system composed by two independent recurrent networks, A and B, having respectively N and M stationary (or quasi-stationary) states, and both densely connected to an output network C. Suppose that the number of neurons in the network C and the number of links from A and from B to C are sufficiently high that each couple of states (S_A, S_B) *of the input networks produces a different state* S_C *of the output network. Then the number of possible output states is* $N \times M$*: Therefore, using the SSM paradigm, C can be represented by a SSM with* $N \times M$ *neurons, and each possible output state can be represented by an output pattern with a single neuron on and all the others off. The two-dimensional SSM can thus be regarded a simplified model of the network described above.*

The input-phrase front layer is single-connected to the input-phrase buffer (InPhB). The input-phrase buffer is also single-connected to itself (self connection). In this way, it can store all words of a phrase and keep them stored until it is cleared by a flush signal.

The connections between the front layer and the back layer (buffer) and the self connections of the back layer are one-by-one connections only for saving memory and computing-time resources. A similar response could be obtained in a framework closer to biological networks by using nearest-neighbor connections.

3 Copy of the input phrase to the working-phrase buffer

Figure 3 shows how the input phrase is copied from the input-phrase buffer (InPhB) to the *working-phrase buffer* (WkPhB). The copy is triggered by the gatekeeper neuron WkFlag, which is fully connected to the *working-phrase front layer* (WkPhFL) and to the *current working* phrase (CurrWkPh). The links from WkFlag to CurrWkPh have negative weight with a very large absolute value, BW (*big weight*). When WkFlag is on, CurrWkPh is cleared. At the same time, the content of InPhB is copied to WkPhFL, which is then copied to the working-phrase buffer, where it is stored thanks to the links between WkPhB and CurrWkPh.

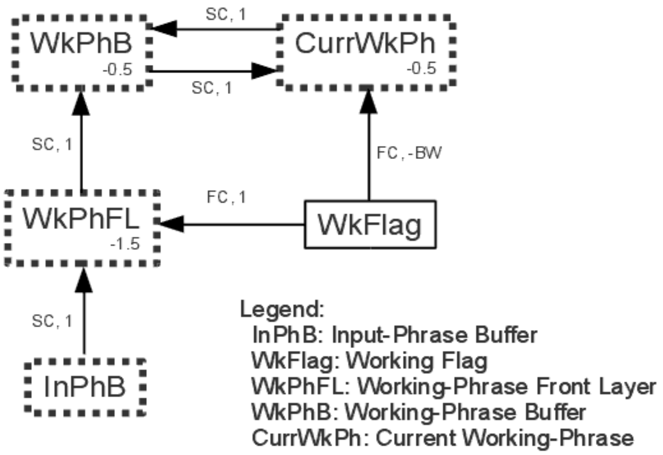

Figure 3. Storage of a word in the working-phrase buffer.

4 Extraction of a word from the working-phrase buffer

Figure 4 represents the architecture used to extract from the working-phrase buffer the word corresponding to the phrase index PhI. WkWfI (working-phrase word-from-index) is a 2DSSM, single-connected to the working-phrase buffer WkPhB and with each column single-connected to PhI. The bias is set in such a way that only the word corresponding to the phrase index PhI is copied from WkPhB to WkWfI. This word is then copied to the current-word buffer CW.

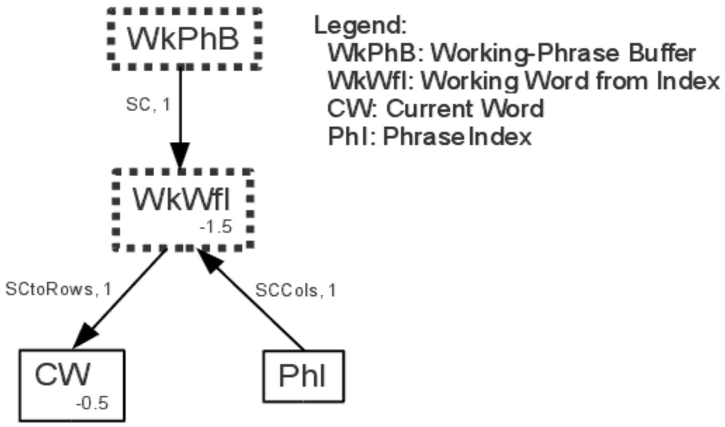

Figure 4. Extraction of the current word from the working-phrase buffer.

5 Copy of a word from the current-word buffer to the word-group buffer

Figure 5 shows how the word in CW is copied to the word-group buffer WGB. This mechanism, together with the one described in the previous paragraph, can be used to extract a group of words from the working-phrase buffer and to store it in the word-group buffer. The architecture is quite similar to that shown in Fig. 2. When the two gatekeeper neurons GetFlag and WGFlag (word-group flag) are on, the word in CW is copied to WGCW (word-group current word) and the index WGI (word-group index) is copied to WGIFL (word-group-index front layer). The 2DSSM WGFL (word-group front layer) is connected to WGCW and to WGIFL in such a way that the couple (word, index) is mapped to the neuron of WGFL located in the row i corresponding to the word-group index and in the column j corresponding to the word. The content of the WGFL is then copied to the word-group buffer (WGB), where it is stored thanks to the self links, until the word-group buffer is cleared by a flush signal.

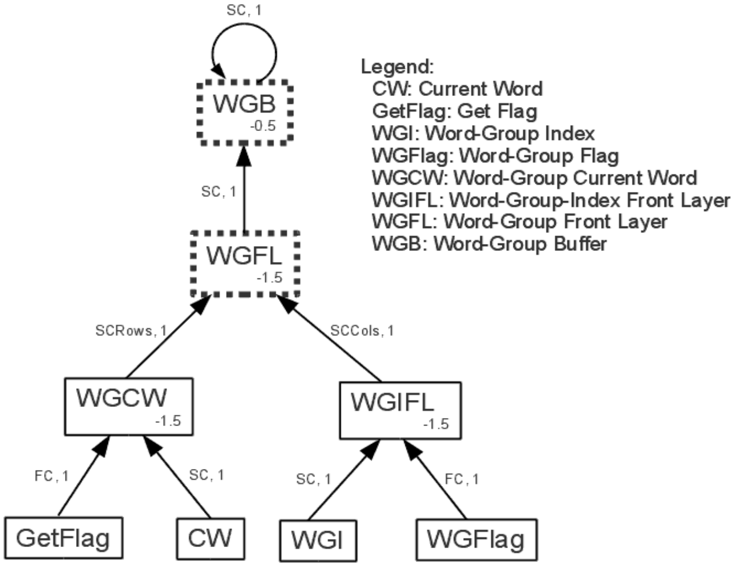

Figure 5. Storage of a word in the word-group buffer.

6 Copy of the word group to the output buffer

Figure 6 shows the architecture that is used to send the current word group to the output. When the gatekeeper neuron OutFlag is in a high-level state, the content of the word-group buffer is copied to OutPhFL (output-phrase front layer) and from there to OutPhB (output-phrase buffer), where it is stored through the self connections until OutPhB is cleared by a flush signal.

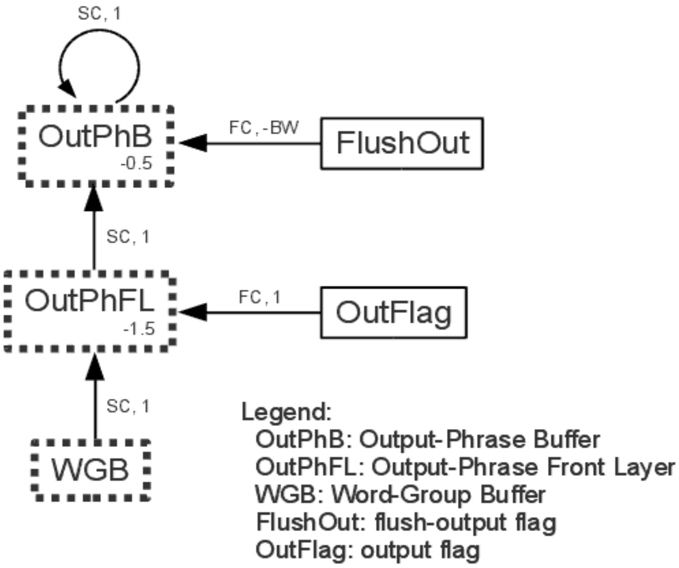

Figure 6. Copy of a word group from the word-group buffer to the output buffer.

7 Memorization of a phrase

Figure 7 represents the architecture used to memorize permanently a phrase. MemPh (memorized phrase) is a SSM used to map all phrases memorized by the system. Every time a new input phrase is acquired by the system, it is mapped to a neuron of MemPh. The neuron update mechanism will be described in a following paragraph. The memorization of a new phrase is triggered by the gatekeeper neuron NewMemPhFlag. When this flag is on, the phrase index in MemPh is copied to RemPhIL (remembered-phrase input layer) and then to RemPh (remembered phrase). RemPh is an associative SSM. The build process is controlled by NewMemPhFlag: when this neuron is on, the link weights from the active neuron of RemPh to WkPhB are updated through the discrete Hebbian learning rule. In this way, the association between the phrase index in MemPh and the phrase is stored permanently in the link weights.

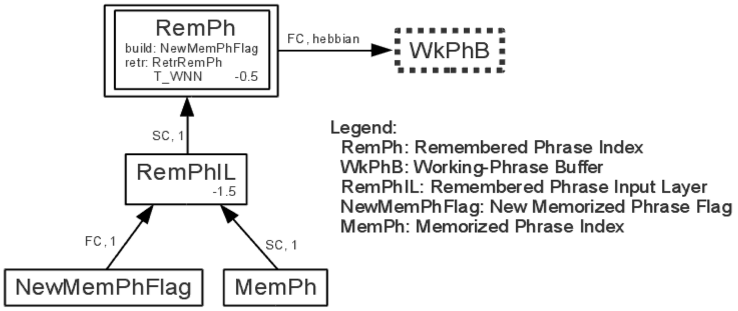

Figure 7. Memorization of the phrase from the working-phrase buffer.

8 Memorization and retrieval of the association between a word group and a phrase

The association between the word group in WGB and the phrase in WkPhB is memorized permanently by the system using the architecture shown in Fig. 8. RemPhfWG is an associative SSM, with input links fully connected to WGB and output links fully connected to RemPh. The build process is controlled by the gatekeeper neuron BuildAs: when this flag is on, a neuron of RemPhfWG is switched to a high-level state through the *winner-take-all* (WTA) rule, and both the weights of the input links from RemPh to this neuron and the weights of the output links form this neuron to WkPhB are updated through the discrete Hebbian learning rule (DHL rule). In this way, the association between the current content of WGB and the current content of RemPh is permanently memorized by the system. The retrieve process is triggered by the gatekeeper neuron RetrAs. The word group in WGB is sent as input to RemPhfWG. The neurons having link weights matching the word group will have the highest activation state, and a single winner is selected among them through the WTA rule. The winner neuron will retrieve the phrase associated to the input word group by using its forcing output links to set the activation state of WkPhB.

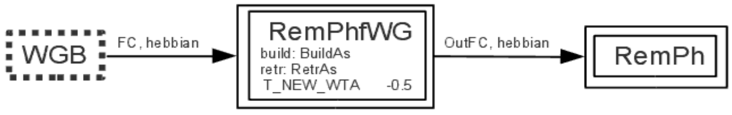

Figure 8. Memorization of the association between the word group and the phrase.

9 Phrase index and word-group index update

Figure 9 represents the architecture used to update the phrase index. This process is controlled by the two gatekeeper neurons CurrPhIFlag (current phrase-index flag) and NextPhIFlag (next phrase-index flag). Those neuron are mutually exclusive: when one is in a high-level state, the other one must necessarily be in a low-level state. When CurrPhIFlag is on, the current phrase index is copied from PhI to CurrPhI, and then it is copied back to PhI. In this way, PhI can hold the current phrase index. On the other hand, when NextPhIFlag is on the current phrase index is copied from PhI to NextPhI. The connection from NextPhI to PhI is a single connection displaced by one neuron, i.e. each neuron of NextPhI is connected to the neuron of PhI located in the following position. In this way, when NextPhIFlag is on PhI will be updated to the next phrase index. If neither CurrPhI nor NextPhI are selected PhI will be cleared. The links from NextPhIFlag and NextPhI to the first neuron of PhI are used to initialize the phrase index in PhI to 1 when PhI is initially empty and NextPhI is selected.

The word-group index is updated using the same type of architecture, as shown in Fig. 10. The update is controlled by the two gatekeeper neurons CurrWGIFlag (current word-group-index flag) and NextWGIFlag (next word-group-index flag). When CurrWGIFlag is on, WGI holds the current word-group index. On the other hand, when NextWGIFlag is on, WGI is updated to the next word-group index. If neither CurrWGI nor NextWGI are selected WGI will be cleared.

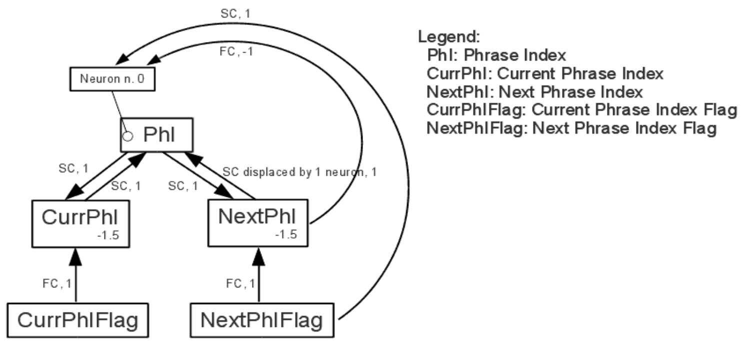

Figure 9. Phrase index update.

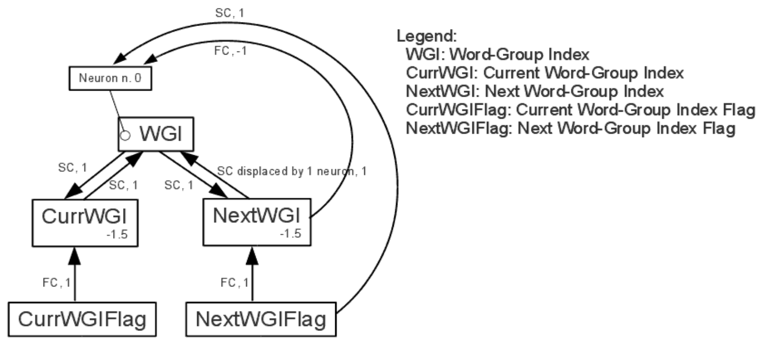

Figure 10. Word-group index update.

10 Memorized phrase and remembered phrase index update

The architecture used to update the index for phrase memorization, shown in Fig. 11, is quite similar to that used for the phrase index update described previously. The update is controlled by the gatekeeper neuron NewMemPhFlag. When NewMemPhFlag is off, MemPh holds the current memorized phrase index. On the other hand, when NewMemPhFlag is on, MemPh is updated to the next memorized phrase index.

The system has the ability to retrieve sequentially the memorized phrases. After a memorized phrase is remembered, for instance by the association mechanism described previously, the system can retrieve the next memorized phrase through the architecture represented in Fig. 12. When the gatekeeper neuron CurrRemPhFlag (current remembered phrase flag) is on, the system holds the memorized phrase index

stored in RemPh. On the other hand, when the gatekeeper neuron NextRemPhFlag is on, RemPh will be updated to the next memorized phrase index.

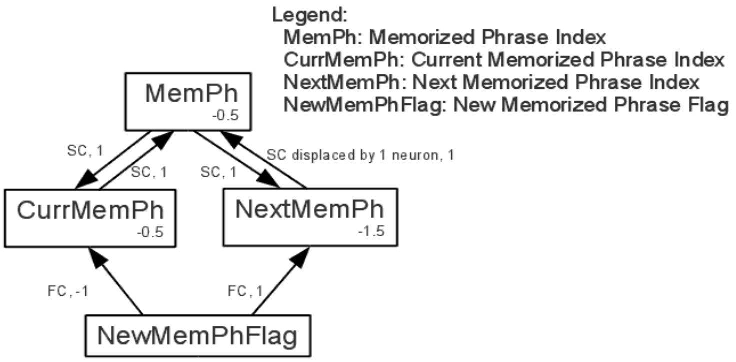

Figure 11. Memorized-phrase-index update

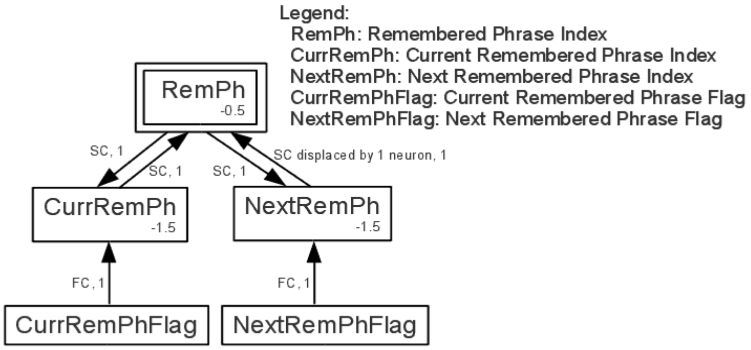

Figure 12. Remembered-phrase-index update.

11 Equal-words vectors

The system is able to recognize whether a phrase buffer contains words that are equal to the word currently stored in CW, or to the words stored in the word-group buffer WGB. The equality among words is represented by binary vectors. For instance, the equality among the words in the input phrase buffer InPhB and the word in CW is represented by the state of the neurons in the input-equal-words (InEqW) SSM. If the i-th word of the input phrase buffer is equal to the word in CW, then the i-th neuron of InEqW will be on, otherwise it will be off. Figure 13 represents the architecture used to produce the equal-words vector InEqW.

In a similar way, the words in the phrase buffers are compared against the words in the word-group buffer. The current version of the system considers only the first four words of the word-group buffer. This simplification is justified by the consideration that the word-group buffer should not contain too many words. For instance, the equality among the words in the input phrase buffer InPhB and the first word in WGB is represented by the state of the neurons in the input-equal-word-group-word-1 (InEqWGW1) SSM. Figures 13 through 17 illustrate the architectures used to produce the equal-word vectors for the input phrase buffer, the working phrase buffer, the word-group buffer and the output buffer.

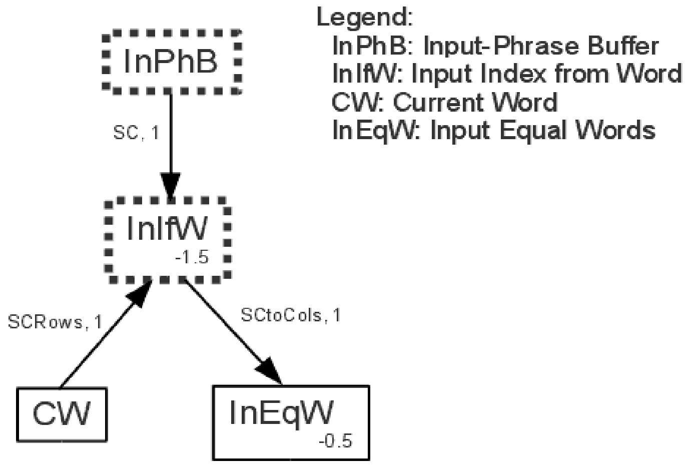

Figure 13. Determination of the input-phrase-equal-words vector from the input-phrase buffer and from the current-word buffer.

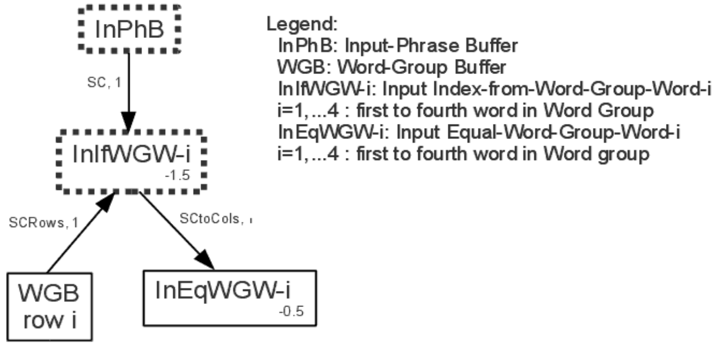

Figure 14. Determination of the input-phrase-equal-words matrix from the input-phrase buffer and from the word-group buffer.

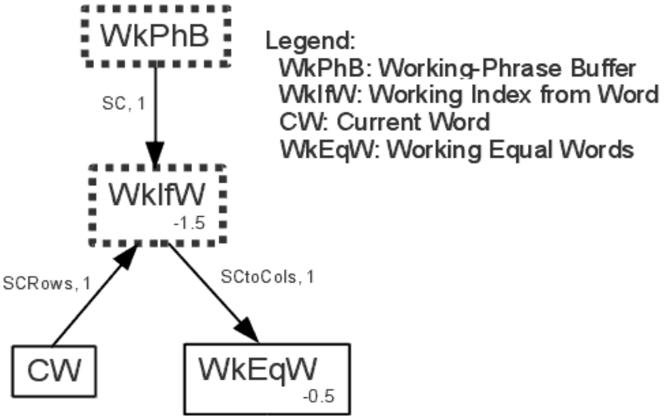

Figure 15. Determination of the working-phrase-equal-words vector from the working-phrase buffer and from the current-word buffer.

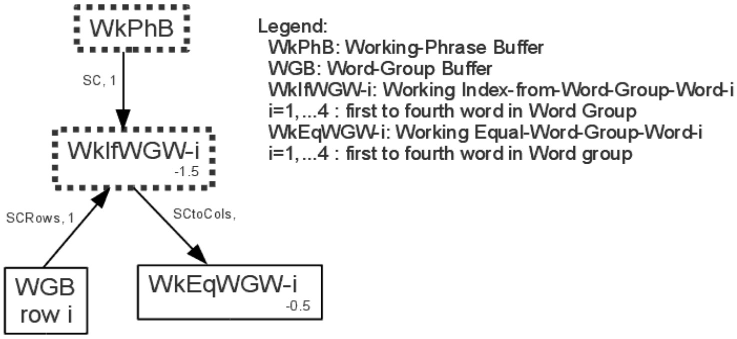

Figure 16. Determination of the working-phrase-equal-words matrix from the working-phrase buffer and from the word-group buffer.

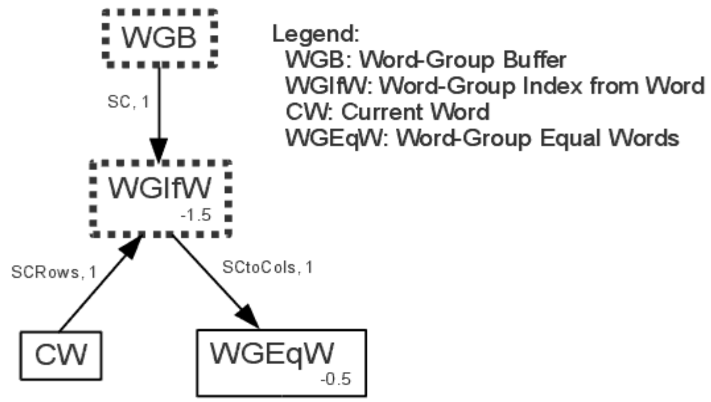

Figure 17. Determination of the word-group-equal-words vector from the word-group buffer and from the current-word buffer.

12 Previous working phrase, previous word-group and previous equal-word vectors

Before extracting a word group from the working phrase, the system clears the current content of the word-group buffer. Similarly, before retrieving a phrase through the association mechanism, the system clears the current content of the working phrase buffer. The learning ability of the system can be improved by preserving the information stored in the word-group buffer and in the working-phrase buffer before clearing them. This information is copied in dedicated SSMs , i.e. a previous-working-phrase buffer, a previous-word-group buffer and previous-equal-words vectors.

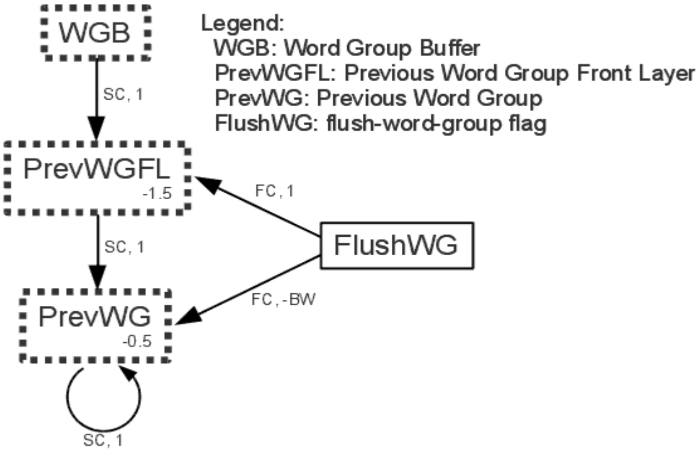

Figure 18. Copy of the word group from the word-group buffer to the previous-word-group buffer.

Figure 18 shows the architecture used to copy the word group from the word-group buffer to the previous-word-group buffer before flushing it. The intermediate SSM PrevWGFL (previous-word-group front layer) is used to decouple the previous word group from the current word group, and to allow the copy only when a flush signal is sent to the word group. It is important to outline that PrevWGFL must be updated *before* WGB, so that the content of WGB is copied before it is flushed. For the same reason, PrevWG must be updated before PrevWGFL.

When a FlushWG signal is sent, the content of WGB is copied to PrevWGFL and PrevWG is cleared. In the next update, when the FlushWG signal is off, the content of PrevWGFL is copied to PrevWG, where it is stored thanks to the self links.

Similar architectures are used to fill the previous-working-phrase buffer and the previous-equal-words vectors. Those architectures are shown in Figs. 19 through 21.

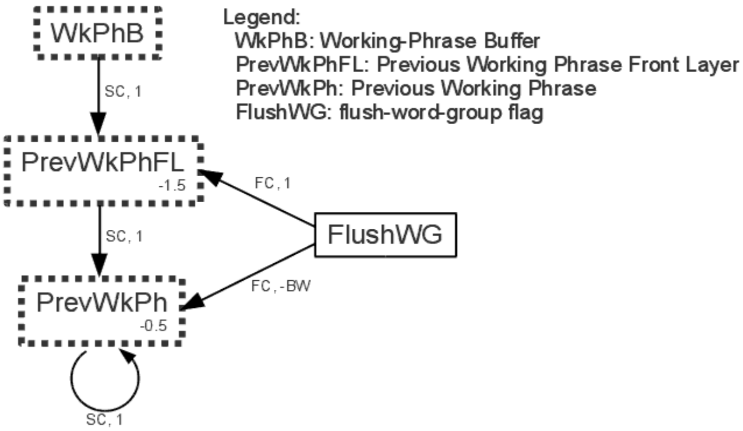

Figure 19. Copy of the phrase from the working-phrase buffer to the previous-working-phrase buffer.

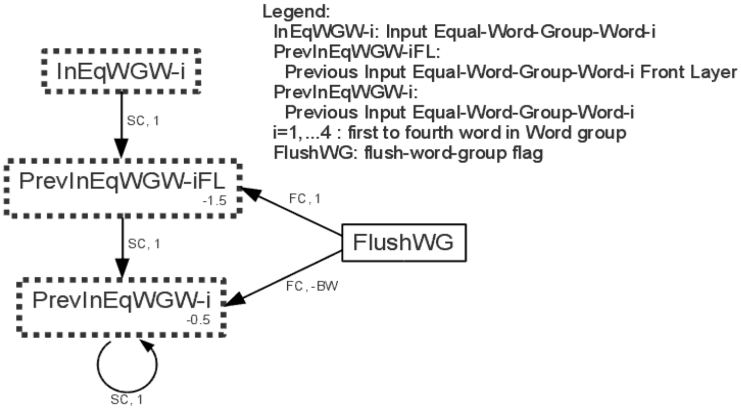

Figure 20. Copy of the input-equal-word-group matrix to the previous-input-equal-word-group buffer.

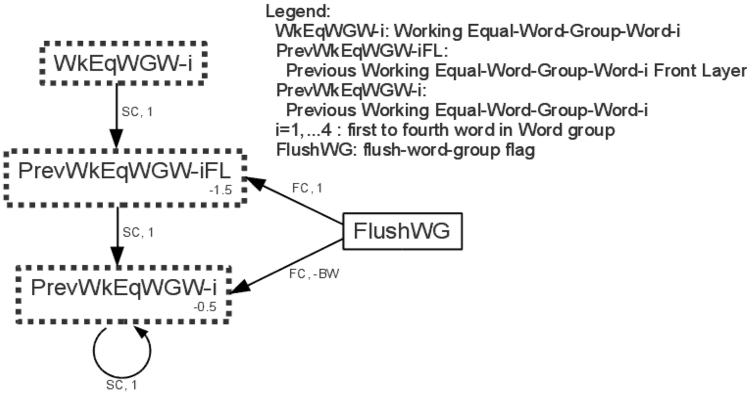

Figure 21. Copy of the working-phrase-equal-word-group matrix to the previous-working-phrase-equal-word-group buffer.

13 Set and retrieve the starting phrase of a context

The system is able to mark the starting phrase of a context and to retrieve it from any other phrase of the same context. Figure 22 shows how a memorized phrase is marked as starting phrase. When the gatekeeper neuron StartPhFlag is switched on, the memorized-phrase index is copied to StartPhIL (starting-phrase input layer) and then to StartPh (starting phrase). On the other hand, when StartPhFlag is off StartPh holds its value thanks to the bidirectional connections to CurrStartPh (current starting phrase).

The system should be able to retrieve the starting phrase from any phrase of the same context. In order to do this, each memorized phrase must be associated to the corresponding starting phrase. Figure 23 show how this association is built. When a new phrase is memorized, the gatekeeper neuron NewMemPhFlag is on; the index of the starting phrase of the current context is copied from StartPh to StartWkPhIL (strarting-working-phrase input layer) and then to StartWkPh (starting working phrase). During this stage RemPh holds the memorized phrase index, because NewMemPhFlag is on, as shown in Fig. 7. The output links from the active neuron of RemPh to StartWkPh are updated by the discrete Hebbian learning rule. In this way the association between the current phrase and the starting phrase of the current context is stored permanently in the links.

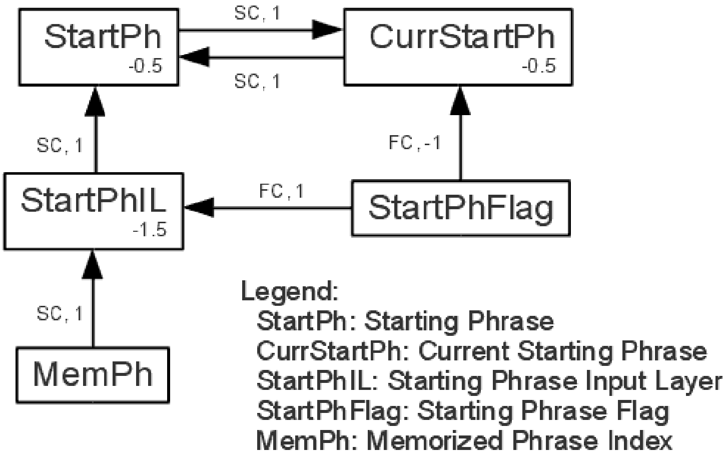

Figure 22. Architecture used for setting the starting phrase of a context.

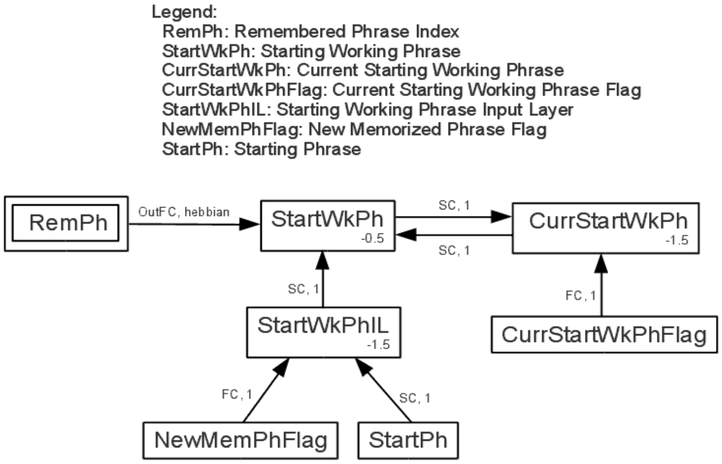

Figure 23. Association between a memorized phrase and the starting phrase of the same context.

When the gatekeeper neuron RetrRemPh is on, StartWkPh is retrieved thanks to the forcing links of RemPh, as shown in Fig. 23. On the other hand, when the action flag CurrStartWkPhFlag is on, StartWkPh holds its value thanks to the bidirectional connections to CurrStartWkPh (current starting working phrase).

Figure 24 illustrates how the starting phrase of the working-phrase context is retrieved. The retrieval is triggered by the gatekeeper neuron RemStartPhFlag (remember-starting-phrase flag). When this flag is on, the content of StartWkPh is copied to RemStartPh (remembered starting phrase) and then to RemPh. The starting phrase is thus retrieved in the working-phrase buffer thanks to the forcing output links from RemPh to WkPhB.

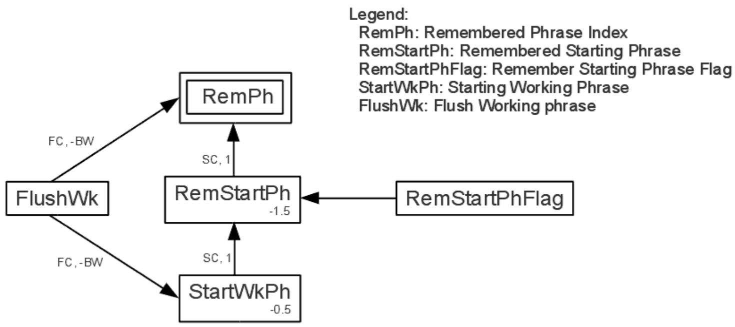

Figure 24. Retrieval of the starting phrase of the current working phrase context.

14 The goal stack

When the working phrase and/or the word group are associated to some important task that cannot be performed immediately, they can be inserted in the top of a goal stack through an architecture that is similar to that described in Sect. 3. This process is controlled by the gatekeeper neuron SetGoalFlag, as shown in Figs. 25 and 26. When this neuron is on the working phrase and the word group are copied to the goal phrase and to the goal word group, respectively.

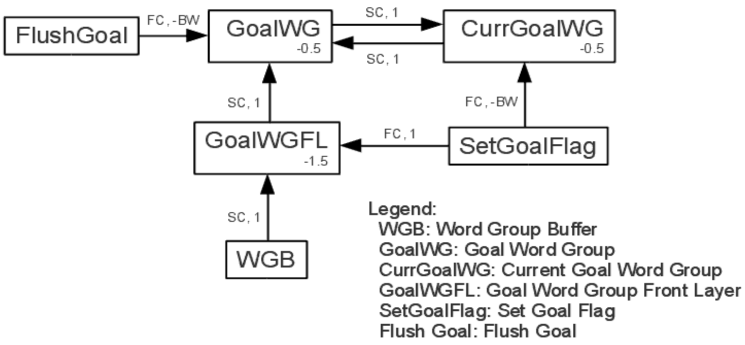

Figure 25. Insertion of the current word group in the goal stack.

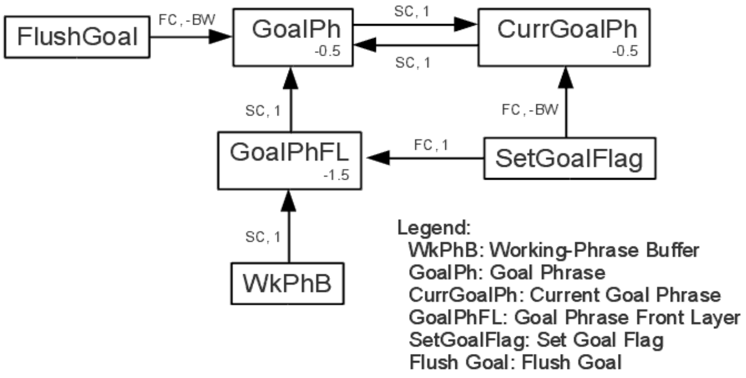

Figure 26. Insertion of the working phrase in the goal stack.

If necessary, the working phrase can be extracted from the top of the goal stack. The extraction is controlled by the gatekeeper neuron GetGoalFlag, as shown in Fig. 27.

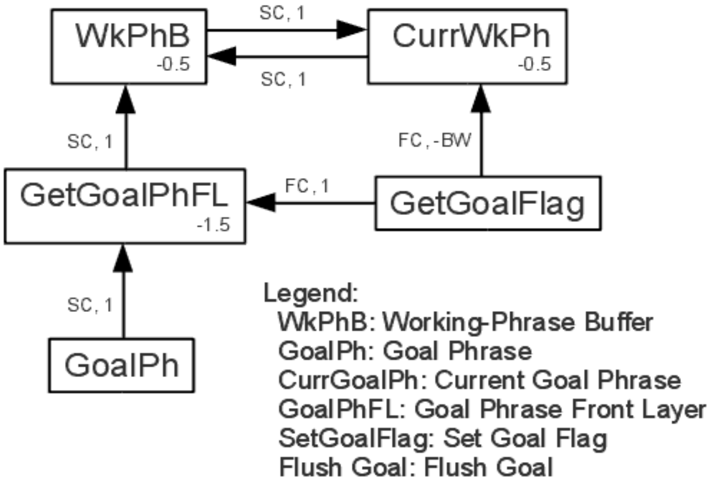

Figure 27. Extraction of the goal phrase from the top of the goal stack to the working phrase.

The goal stack can hold simultaneously up to 10 phrases and word groups. The insertions and the extractions follow the LIFO (last in, first out) order. The architecture used for storing more phrases and word groups is similar to that described in Sect. 9. A goal index, stored in the SSM GoalI, can be

increased or decreased through the gatekeeper neurons NextGoalIFlag and PrevGoalIFlag, respectively, as shown in Fig. 28.

Figure 36: Goal Index Update

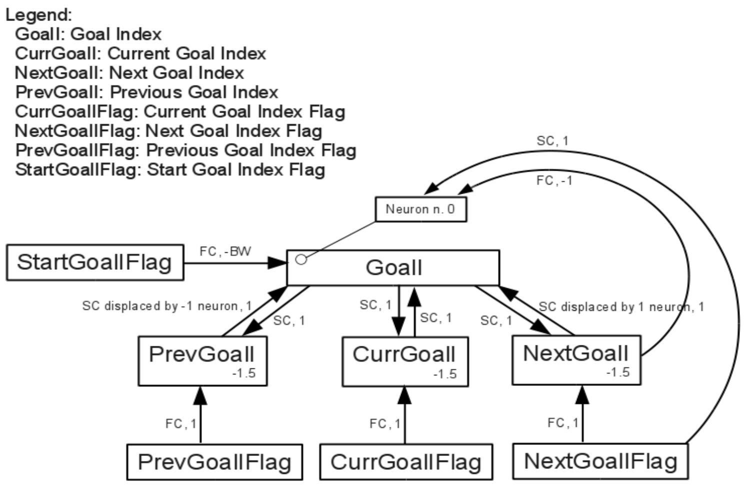

Figure 28. Architecture used to control the goal-index update.

The goal phrase and the goal word group corresponding to the index GoalI are memorized and retrieved through the associative SSM GoalMem, as shown in Fig. 29. The memorization process is controlled by the gatekeeper neuron BuildGoal: when this flag is on, the weights of the output links from the active neuron of GoalMem to GoalWG and GoalPh are updated through the DHL rule. The extraction process is triggered by the gatekeeper neuron RetrGoal. The goal phrase and the goal word group associated to the current goal index are retrieved by using the forcing output links of GoalMem to set the activation states of GoalPh and GoalWG.

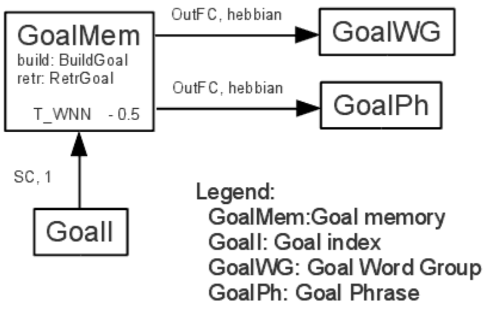

Figure 29. Retrieval of the goal phrase and goal word group corresponding to the goal index GoalI from the goal memory.

15 Goal equal-words vectors

The system is able to recognize whether the working phrase or the goal phrase buffer contains words that are equal to the words stored in the goal word group GoalWG. Figures 30 and 31 illustrate the architectures used to produce the equal word vectors, which are analogous to those described in Sect. 11.

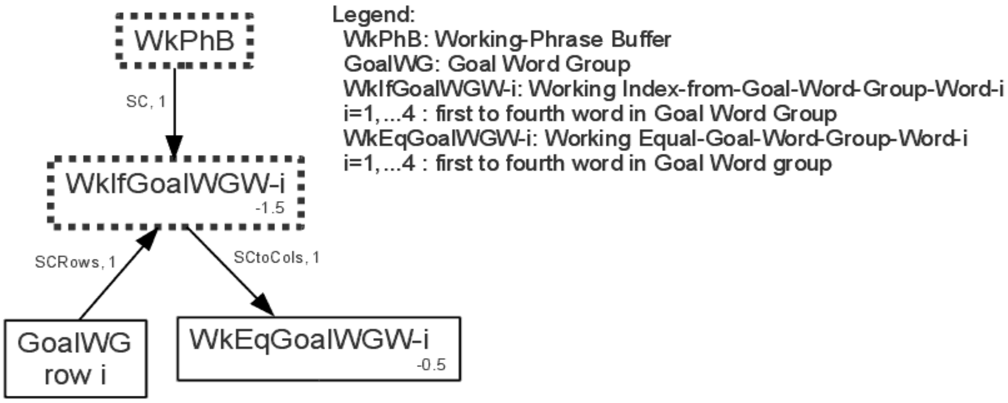

Figure 30. Determination of the working-phrase-equal-goal-word-group matrix from the working-phrase buffer and from the goal word group.

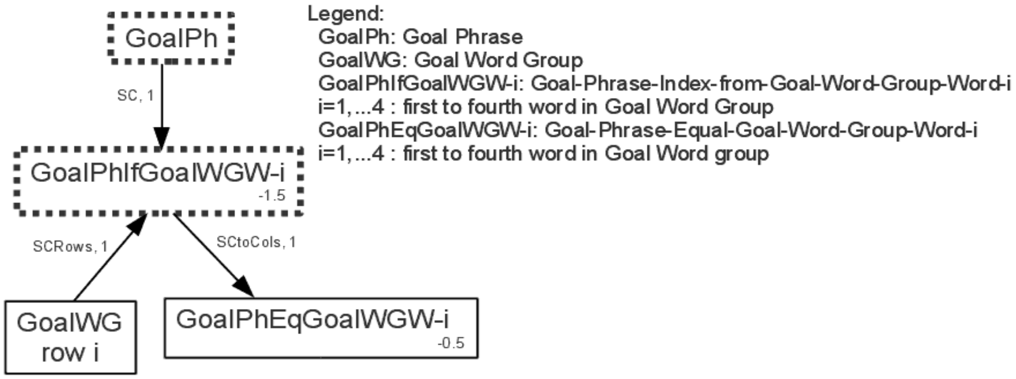

Figure 31. Determination of the goal-phrase-equal-goal-word-group matrix from the goal phrase and from the goal word group.

16 Action and gatekeeper neurons

The system execute commands by means of special neurons called *action neurons*. Each action neuron correspond to a command. The action neurons set the values of the gatekeeper neurons, which control the system operation, as discussed in the previous sections.

The action neurons are divided in 3 groups: acquisition-action neurons, elaboration-action neurons and reward-action neurons. All types of actions (acquisition, elaboration and reward actions) require a single complete update of the system to be executed. The acquisition actions are performed during the acquisition of the input phrases (acquisition operating mode) and during the process of building the association between word groups and phrases (association operating mode). The elaboration actions are performed during the exploration phases and during the exploitation operating modes.

The acquisition-action neurons, the elaboration-action neurons and the reward-action neurons are grouped in three SSMs, called AcqAct, ElAct and RwdAct, respectively, while the gatekeeper neurons are grouped in a SSM called ActFlags. AcqAct, ElAct and RwdAct are fully connected to ActFlags. The link weights determine which action flags are activated and which ones are deactivated by the actions. Figure 32 shows the connections between the action-neurons SSMs and the gatekeeper-neurons SSM.

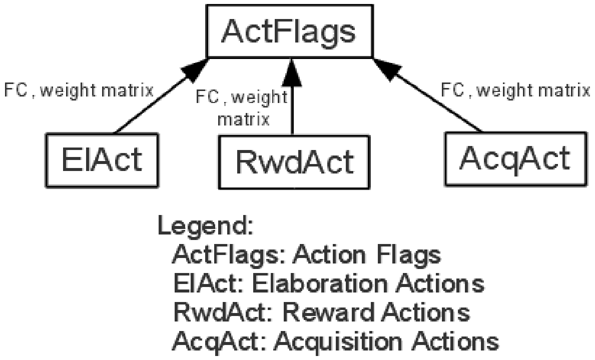

Figure 32. Connections between the action neurons SSMs and the gatekeeper-neurons SSM.

The way how gatekeeper neurons are used in the architecture to control the system operations was described in the previous sections. Below is the list of the acquisition, elaboration and reward actions, with a short description of their effect.

Acquisition actions:

- *FLUSH*: clears the content of all phrase and word-group buffers;
- *ACQ_W*: acquires a word from the word-map front layer to the input-phrase buffer.
- *NEXT_AS_W*: copies a word from the working-phrase buffer to the word-group buffer and points to the next word;
- *BUILD_AS*: stores the association between the current word group in WGB and the current working phrase;
- *MEM_PH*: memorizes the current working phrase;
- *SET_START_PH*: sets the current working phrase as the starting phrase of the context.

Elaboration actions:

- *NULL_ACT*: null action;
- *FLUSH_WG*: clears the content of the word-group buffer;
- *W_FROM_WK:* initializes the phrase index (PhI) to zero, to prepare the extraction of words from the working-phrase buffer;
- *W_FROM_IN:* initializes the phrase index (PhI) to zero and copies the input phrase to the working phrase buffer;
- *NEXT_W*: skips a words of the working phrase buffer;

- *GET_W*: copies a word from the working phrase buffer to the word-group buffer;
- *RETR_AS*: retrieves a phrase associated to the word group by the association mechanism.
- *FLUSH_OUT*: clears the content of the output buffer;
- *WG_OUT*: copies the content of the word-group buffer to the output buffer;
- *GET_NEXT_PH*: retrieves sequentially phrases belonging to the same context;
- *GET_START_PH*: retrieves the starting phrase of the same context of the working phrase;
- *CONTINUE*: labels the end of a state-action sequence that can receive a partial reward;
- *DONE*: labels the end of a state-action sequence that can receive a (conclusive) reward;
- *PUSH_GL*: copies the working phrase to the goal stack;
- *DROP_GL*: delete a phrase from the goal stack;
- *GET_GL_PH*: copies a phrase from the goal stack to the working phrase buffer;
- *SNT_OUT*: sends to the output the next part of the working phrase, after the current word, and all subsequent phrases of the same context until the end of the context itself.

Reward actions:

- *NULL_RWD*: null action;
- *STORE_ST_A*: stores the current state and action in the state-action memory and increases the state-action index;
- *START_ST_A*: resets the state-action index to the beginning of the state-action sequence;
- *RETR_ST_A*: retrieves the state and action from the state-action memory and increases the state-action index;
- *RWD_ST_A*: rewards the last action by a change in the link weights of the state-action association SSM;
- *CHANGE_ST_A*: stores the current state and action in the state-action memory without increasing the state-action index;
- *GET_ST_A*: retrieves the state and action from the state-action memory without increasing the state-action index;
- *RETR_EL_A*: uses the state-action association SSM to find the best action associated to the current state;
- *STORE_SAI*: stores the current state-action index in a buffer;
- *RETR_SAI*: retrieves the state-action index from the buffer where it was stored;

- *RETR_ST*: retrieves only the state from the state-action memory, set the action to a *NULL* value and increases the state-action index;

17 Memorization and retrieval of a state-action sequence

When an exploration phase leads to a target output, the human interlocutor can use the interface to trigger a reward signal, which puts the system in the reward operating mode. In this mode the system retrieves the state-action sequence that yielded the reward, and memorizes the association between each state of the sequence and the corresponding action. The system can memorize up to 300 states and actions, which are indexed by the SSM StActI (state-action index). The architecture used to reset and to increase StActI, which is illustrated in Fig. 33, is analogous to that described in Sect. 9.

The state-action sequence is memorized and retrieved through the associative SSM StActMem, as shown in Fig. 34. When the gatekeeper neuron BuildStAct is on, the association between the index StActI and the state and action are memorized as a change of the output links of StActMem through the DHL rule. On the other hand, when the action flag neuron RetrStAct is on, the state and action corresponding to the index StActI are retrieved.

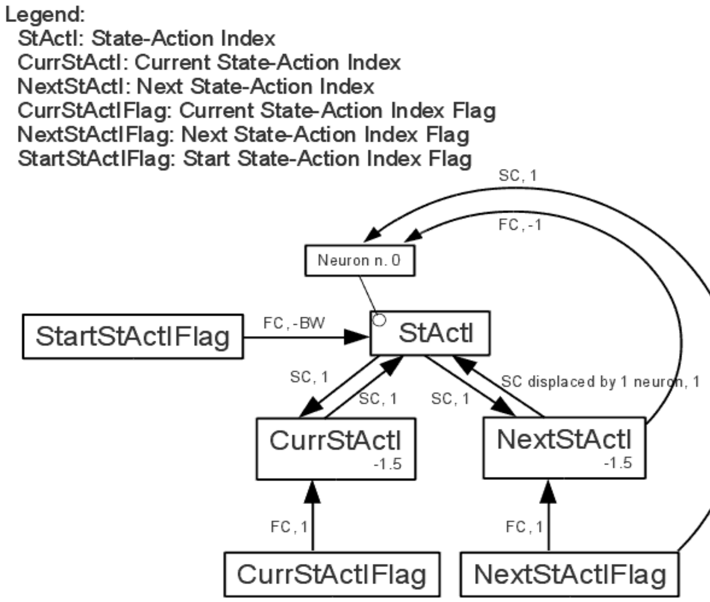

Figure 33. Architecture used for the state-action index update.

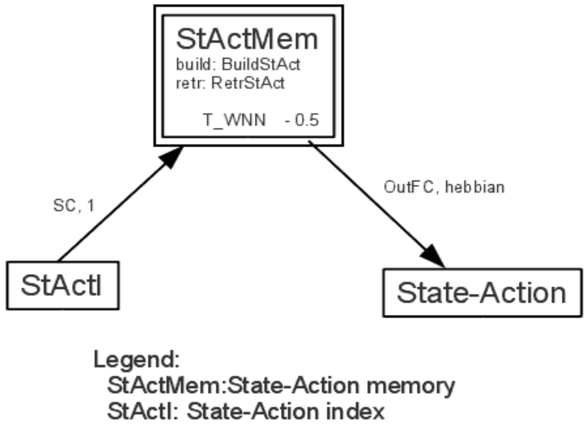

Figure 34. Retrieval of the state and action corresponding to the index StActI of the state-action sequence memorized by StActMem.

18 State-Action association

As discussed in the previous section, during the reward phase the state-action sequence that yielded the reward is retrieved. The association between each state of the sequence and the corresponding action is memorized through the associative SSM ElActfSt. This process is controlled by the gatekeeper neuron BuildElActfSt, as shown in Fig. 35. When this neuron is on, the system state is

mapped to a previously unused neuron of ElActfSt through the WTA rule, and both the links from the state to the winner neuron of ElActfSt and the links from this neuron to ElAct are updated through the DHL rule.

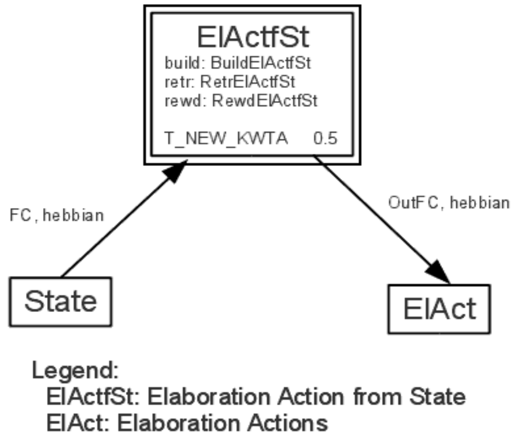

Figure 35. State-action association architecture.

During the exploitation phase, ElActfSt associates an elaboration action to the system state. This process is triggered by the gatekeeper neuron RetrElActfSt. ElActfSt is updated through the k-winner-take-all (KWTA) rule. On the other hand, ElAct is updated through the (one) WTA rule. In this way, ElAct selects a single action, the one that is more represented among the outputs of the k winner neurons of ElActfSt.

19 Operating-modes subnetworks

The five operating modes are controlled by specialized subnetworks, which have been designed through the concept of finite state machine. For each operating mode, the design was made in four steps: first we wrote its high level implementation using C-like pseudocode. Second, the high level pseudocode was translated to low-level pseudocode, consisting of numbered instructions; each of those instructions executes one of the actions listed in Sect. 16, an unconditional jump to another instruction (represented by a *goto* statement) or a conditional jump. Third, the low-level pseudocode was used to

design the block-diagram of a finite state machine, with each node corresponding to an instruction of the pseudocode. Finally, the finite-state machine was implemented in a neural network; each neuron corresponds to a node of the finite state machine, i.e. to an instruction of the low-level pseudocode. The actions are executed through links between those instruction neurons and the corresponding action neurons. Neurons corresponding to consecutive instructions are linked to each other. Similarly, unconditional jumps are achieved through straight links between the instruction neurons. Conditional jumps are implemented using intermediate neurons, as shown in the example in Fig. 36: when the gatekeeper neuron goto_B_flag is on, the intermediate neuron A_goto_B is set to a high-level state, therefore the neuron B is activated. On the other hand, if goto_B_flag is off, A_goto_C is set to a high-level state and thus the neuron C is activated.

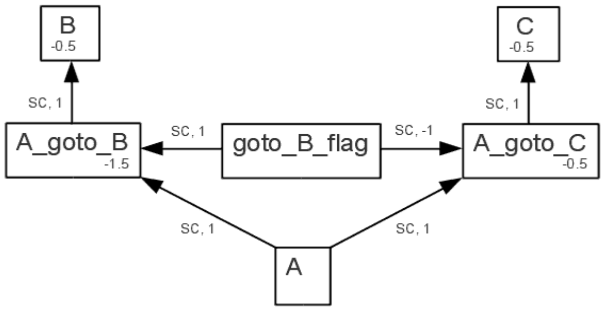

Figure 36. Implementation of a conditional jump at a neural level.

The high-level pseudocode, the low-level pseudocode and the block diagram of the five operating modes are shown below.

19.1 Acquisition

High-level pseudocode:

```
FLUSH;
do {
    ACQ_W;
} while (!BlankWFlag);
W_FROM_WK;
FLUSH_WG;
```

FLUSH_WG; // this instruction is used twice to clear also the previous word group

Low-level pseudocode:

10 FLUSH;

20 ACQ_W;

30 if (! BlankWFlag) goto 20;

40 W_FROM_WK;

50 FLUSH_WG;

60 FLUSH_WG; // this instruction is used twice to clear also the previous word group

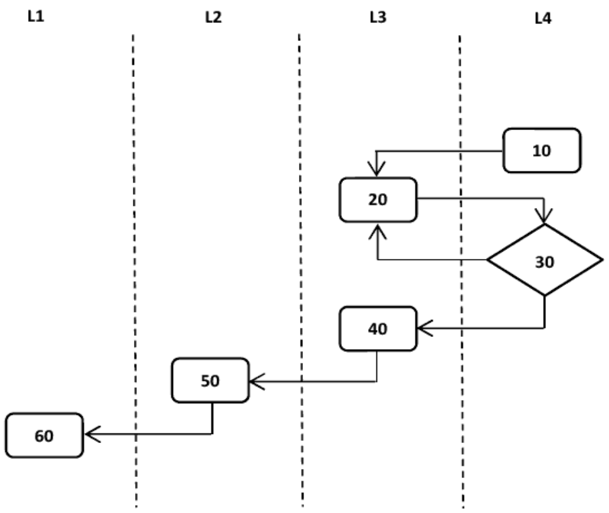

Figure 37. Block-diagram of the acquisition operating mode.

19.2 Association

High-level pseudocode:

SkipW=0;

if (SetStartPhFlag) SET_START_PH;

else MEM_PH;

W_FROM_WK;

while (!EndSkipWFlag && !BlankSkipWFlag) {

    do {

        NEXT_AS_W;

```
    } while (!PhiEqSkipWFlag);
    FLUSH_WG;
    do {
        BUILD_AS;
        NEXT_AS_W;
    } while (!EndWordGroupFlag);
    SkipW++;
    W_FROM_WK;
}
FLUSH_WG;
FLUSH_WG; // this instruction is used twice to clear also the previous word group
```

Low-level pseudocode:

```
10 SkipW=0;
    if (SetStartPhFlag) SET_START_PH;
    else MEM_PH;
20 W_FROM_WK;
30 if (EndSkipWFlag || BlankSkipWFlag) goto 130;
40 NEXT_AS_W;
50 if (!PhiEqSkipWFlag) goto 40;
60 FLUSH_WG;
70 BUILD_AS;
80 NEXT_AS_W;
90 if (!EndWordGroupFlag) goto 70;
100 SkipW++;
110 W_FROM_WK;
120 goto 30;
130 FLUSH_WG;
140 FLUSH_WG; // this instruction is used twice to clear also the previous word group
```

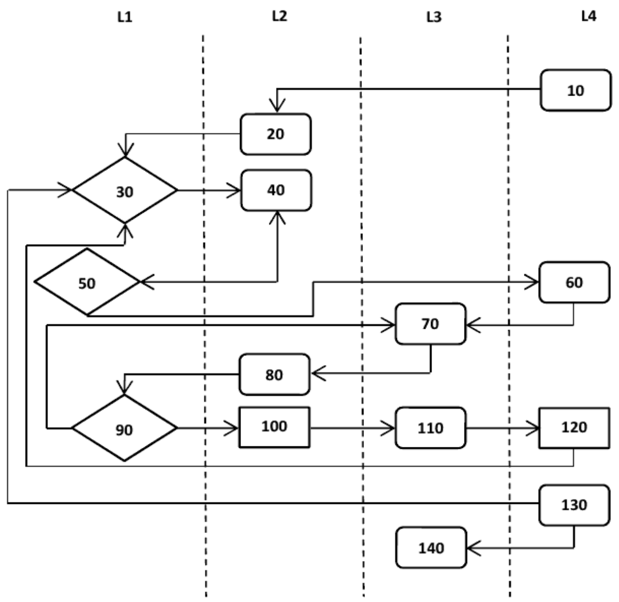

Figure 38. Block-diagram of the association operating mode.

19.3 Exploration

High-level pseudocode:

```
if (WGTargetFlag && (EPhaseI==3 || EPhaseI==4) EPhaseI=1;
if (EPhaseI==0) StEPhaseI=1;
else StEPhaseI=EPhaseI;
IterI=0;
do {
    40 on (EPhaseI+1) goto 50, 100, 240, 310, 340;
    if (EPhaseI==0) {
        START_ST_A;
        W_FROM_IN;
        EXPL_APPR;
        EPhaseI=1;
    }
    if (EPhaseI==1) {
        N1=IntRnd(1,5); // random number between 1 and 5
```

```
    N2=IntRnd(1,5); // random number between 1 and 5
    GetRndFlag=true;
    W_FROM_WK;
    do {
        NEXT_W;
    } while (!PhiEqN1Flag);
    FLUSH_WG;
    do {
        GET_W;
        NEXT_W;
    } while (!PhiEqN2Flag && !EndWordGroupFlag);
    if (WGTargetFlag) {
        CheckWGFlag=true;
        if (TargetEqWGFlag) break;
        else goto 370;
    }
    else {
        EPhaseI=2;
        continue;
    }
}
else if (EPhaseI==2) {
    RetrAs;
    if (WGTargetFlag) {
        EPhaseI=1;
        continue;
    }
    else {
        CheckWkPhFlag=true;
        if (TargetEqWkPhFlag) break;
```

```
        }
    }
    else if (EPhaseI==3) {
        GET_START_PH;
        CheckWkPhFlag=true;
        if (TargetEqWkPhFlag) break;
    }
    else if (EPhaseI==4) {
        GET_NEXT_PH;
        CheckWkPhFlag=true;
        if (TargetEqWkPhFlag) break;
    }
    EphaseI=StEPhaseI;
    if (EPhaseI==3) StEPhaseI=4;
    if (EPhaseI==4) StEPhaseI=1;
    EXPL_RETRY;
    IterI++;
} while (!EndIterIFlag);
if (MaxIterIFlag) display MaxIterI warning message;
else EXPL_APPR;
```

Low-level pseudocode:

```
10 if (WGTargetFlag && (EPhaseI==3 || EPhaseI==4) EPhaseI=1;
20 if (EPhaseI==0) StEPhaseI=1;
    else StEPhaseI=EPhaseI;
30 IterI=0;
40 on (EPhaseI+1) goto 50, 100, 240, 310, 340;
50 START_ST_A;
60 W_FROM_IN;
70 EXPL_APPR;
```

```
80 EPhaseI=1;
100 N1=IntRnd(1,5); // random number between 1 and 5
    N2=IntRnd(1,5); // random number between 1 and 5
    GetRndFlag=true;
110 W_FROM_WK;
120 NEXT_W;
130 if (!PhiEqN1Flag) goto 120;
140 FLUSH_WG;
150 GET_W;
160 NEXT_W;
170 if (!PhiEqN2Flag && !EndWordGroupFlag) goto 150;
180 if (!WGTargetFlag) goto 210;
190 CheckWGFlag=true;
200 if (TargetEqWGFlag) goto 440;
    else goto 370;
210 EPhaseI=2;
220 goto 410;
240 RetrAs;
250 if (!WGTargetFlag) goto 280;
260 EPhaseI=1;
270 goto 410;
280 CheckWkPhFlag=true;
290 if (TargetEqWkPhFlag) goto 440;
    else goto 370;
310 GET_START_PH;
320 CheckWkPhFlag=true;
330 if (TargetEqWkPhFlag) goto 440;
    else goto 370;
340 GET_NEXT_PH;
350 CheckWkPhFlag=true;
```

360 if (TargetEqWkPhFlag) goto 440;

else goto 370;

370 EphaseI=StEPhaseI;

380 if (EPhaseI==3) StEPhaseI=4;

390 if (EPhaseI==4) StEPhaseI=1;

400 EXPL_RETRY;

410 IterI++;

420 if (!EndIterIFlag) goto 40;

430 *display MaxIterI warning message*; goto 450;

440 EXPL_APPR;

450 end;

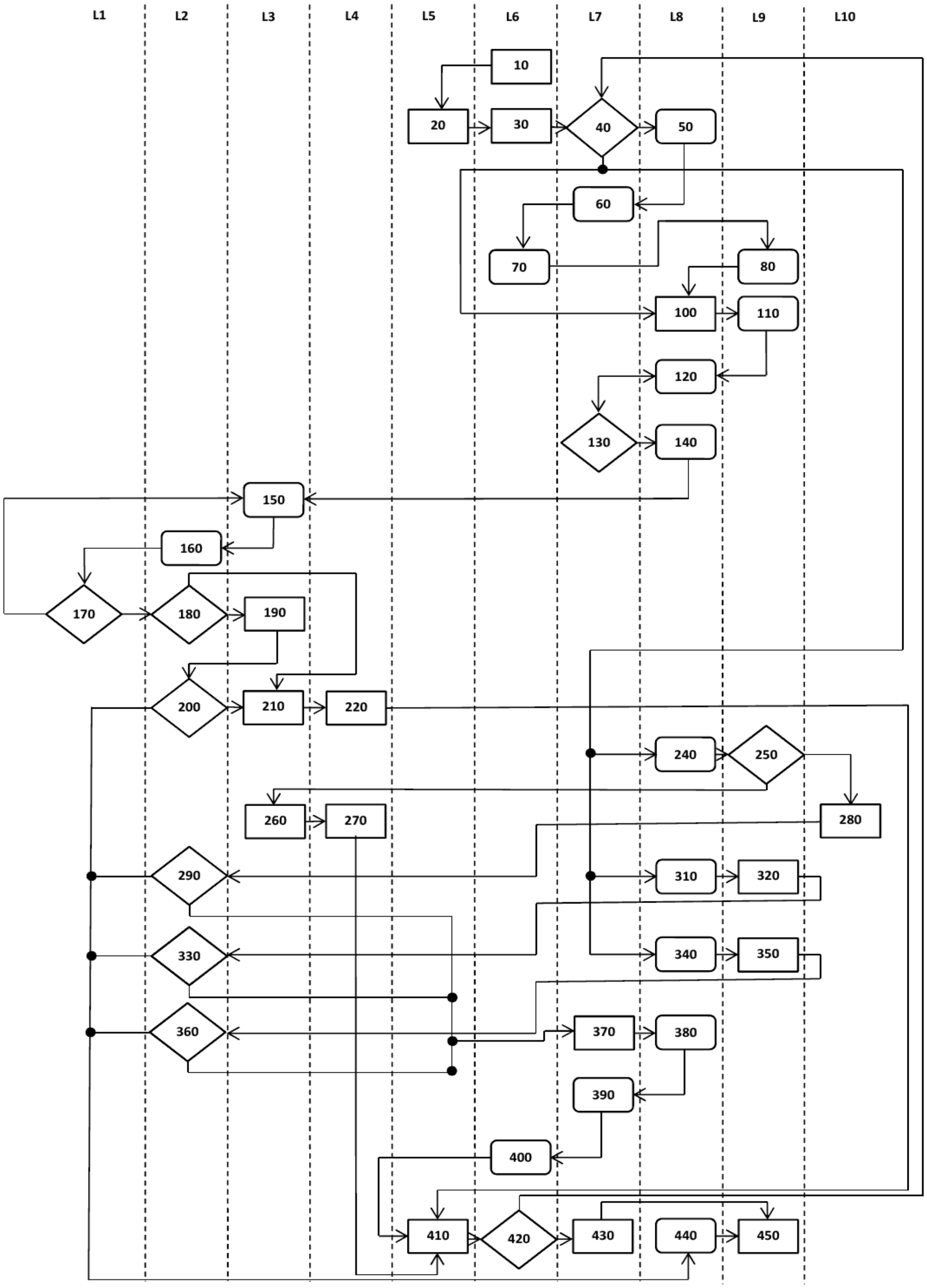

Figure 39. Block-diagram of the exploration operating mode.

19.4 Reward

High-level pseudocode:

```
IterI=0;
STORE_ST_A;
FLUSH_OUT;
STORE_ST_A;
WG_OUT;
STORE_ST_A;
if (PartialFlag) CONTINUE;
else DONE;
STORE_ST_A;
LastStActI=StActI-2;
do {
    START_ST_A;
    IterI++;
    do {
        RWD_ST_A;
    } while (!EndStActIFlag);
} while (!EndIterIFlag);
```

Low-level pseudocode:

```
10 IterI=0;
   STORE_ST_A;
   FLUSH_OUT;
20 STORE_ST_A;
   WG_OUT;
30 STORE_ST_A;
   if (PartialFlag) CONTINUE;
```

else DONE;

40 STORE_ST_A;

50 LastStActI=StActI-2;

60 START_ST_A;

70 IterI++;

80 RWD_ST_A;

90 if (!EndStActIFlag) goto 80;

100 if (!EndIterIFlag) goto 60;

110 end;

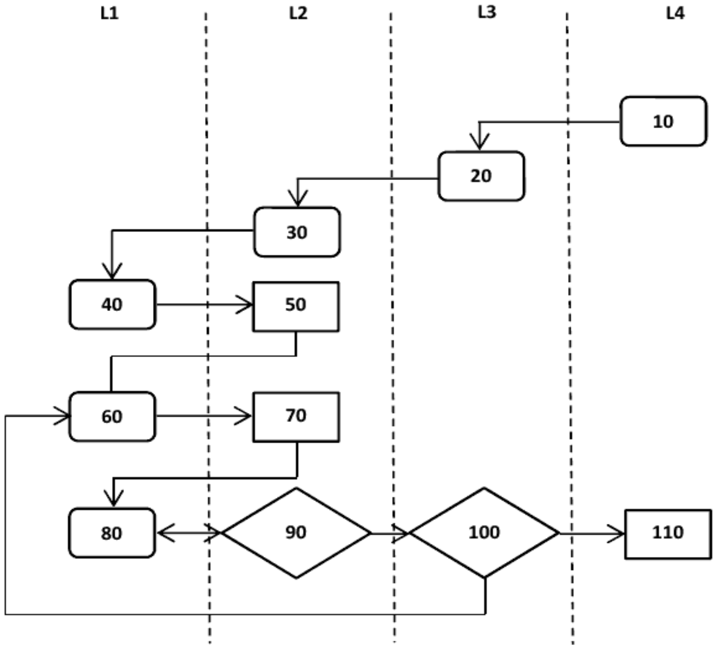

Figure 40. Block-diagram of the reward operating mode.

19.5 Exploitation

High-level pseudocode:

IterI=0;

```
START_ST_A;
STORE_ST_A;
do {
    START_ST_A;
    RETR_ST_A;
    do {
        if (!PrevRetrAsFlag) {
            BestDB=MaxDB;
            BestAct=NULL_ACT;
            STORE_ST_A;
            NRetr=0;
            do {
                RETR_AS;
                ElAct=NULL_ACT;
                RETR_EL_A;
                if (UpdateBestDBFlag) {
                    BestDB=DB;
                    BestAct=ElActFL;
                    CHANGE_ST_A;
                }
                NRetr++;
            } while (!EndNRetrFlag);
            if (BestAct==NULL_ACT) NoRetrFlag=true; break // display warning message
            GET_ST_A;
        }
        else {
            RETR_EL_A;
            BestDB=DB;
            BestAct=ElActFL;
            if (BestAct==NULL_ACT) NoRetrFlag=true; break; // display warning message
```

```
        }
        ElActFL=BestAct; STORE_ST_A;
        if (ElAct==DONE) break;
    } while (!MaxStActIFlag);
    if (MaxStActIFlag) display MaxStActI warning message;
    IterI++;
} while (!EndIterIFlag);
```

Low-level pseudocode:

```
10 IterI=0;
   START_ST_A;
20 STORE_ST_A;
30 START_ST_A;
40 RETR_ST_A;
50 if (!PrevRetrAsFlag) goto 200;
60 BestDB=MaxDB;
   BestAct=NULL_ACT;
70 STORE_ST_A;
80 NRetr=0;
90 RETR_AS;
100 ElAct=NULL_ACT;
110 RETR_EL_A;
120 if (!UpdateBestDBFlag) goto 150;
130 BestDB=DB;
    BestAct=ElActFL;
140 CHANGE_ST_A;
150 NRetr++;
160 if (!EndNRetrFlag) goto 90;
170 if (BestAct==NULL_ACT) NoRetrFlag=true; goto 270 // display warning message
180 GET_ST_A;
```

```
190 goto 230;
200 RETR_EL_A;
210 BestDB=DB;
    BestAct=ElActFL;
220 if (BestAct==NULL_ACT) NoRetrFlag=true; goto 270; // display warning message
230 ElActFL=BestAct; STORE_ST_A;
240 if (ElAct==DONE) goto 270;
250 if (!MaxStActIFlag) goto 50;
260 display MaxStActI warning message;
270 IterI++;
280 if (!EndIterIFlag) goto 30;
290 end;
```

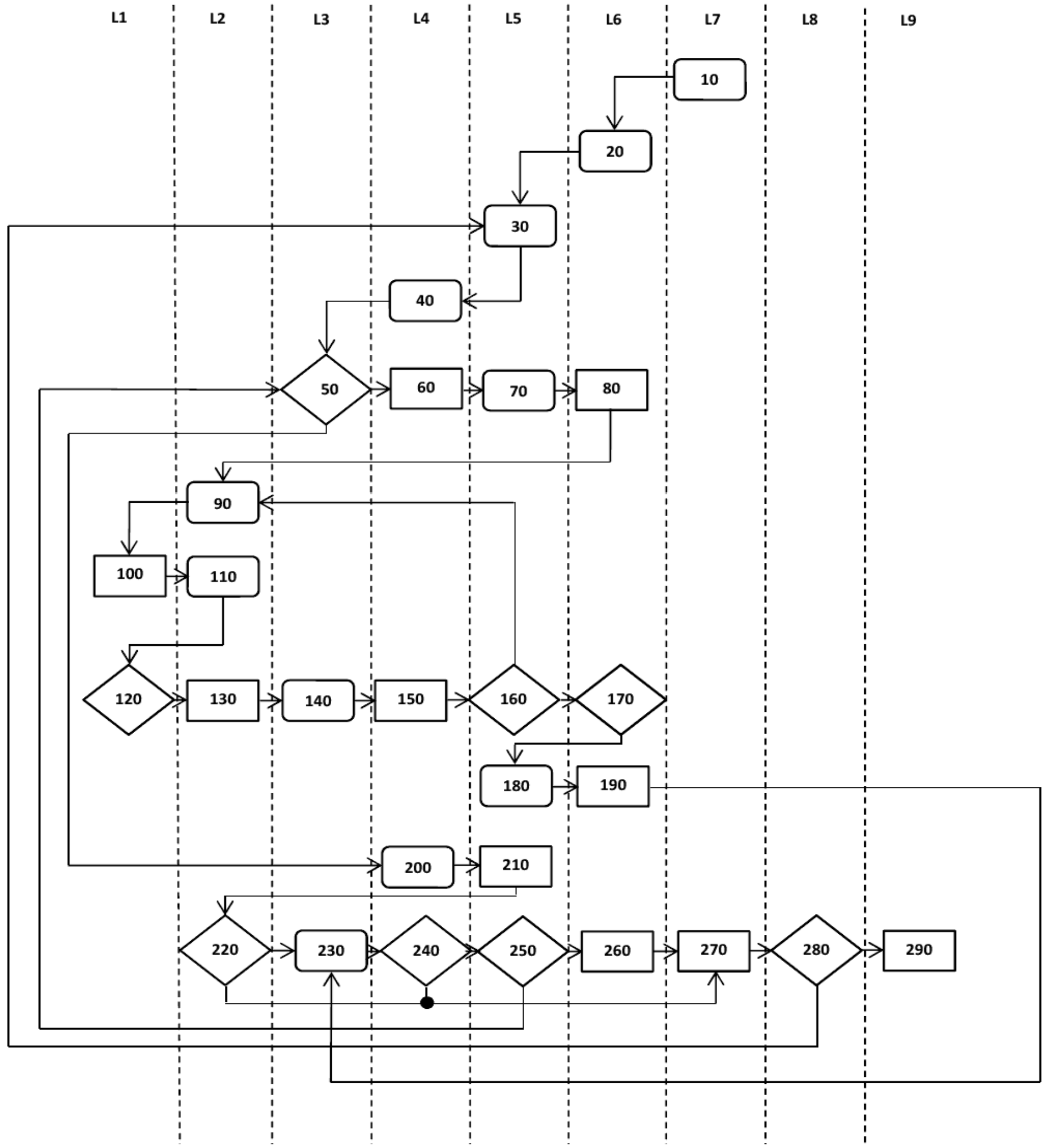

Figure 41. Block-diagram of the exploitation operating mode.
